# Immunoregulatory programs in anti‐*N*‐methyl‐D‐aspartate receptor encephalitis identified by single‐cell multi‐omics analysis

**DOI:** 10.1002/ctm2.70173

**Published:** 2025-01-08

**Authors:** Xinhui Li, Yicong Xu, Weixing Zhang, Zihao Chen, Dongjie Peng, Wenxu Ren, Zhongjie Tang, Huilu Li, Jin Xu, Yaqing Shu

**Affiliations:** ^1^ State Key Laboratory of Biocontrol｜Innovation Center for Evolutionary Synthetic Biology School of Life Sciences Sun Yat‐sen University Guangzhou China; ^2^ Institute of Experimental Cardiology Heidelberg University Heidelberg Germany; ^3^ Department of Neurology The Third Affiliated Hospital of Sun Yat‐sen University Guangzhou China

**Keywords:** anti‐*N*‐methyl‐D‐aspartate receptor encephalitis, autoimmune disease, regulatory program, single‐cell multi‐omics sequencing

## Abstract

**Background:**

Anti‐*N*‐methyl‐D‐aspartate receptor encephalitis (anti‐NMDARE) is a prevalent type of autoimmune encephalitis caused by antibodies targeting the NMDAR's GluN1 subunit. While significant progress has been made in elucidating the pathophysiology of autoimmune diseases, the immunological mechanisms underlying anti‐NMDARE remain elusive. This study aimed to characterize immune cell interactions and dysregulation in anti‐NMDARE by leveraging single‐cell multi‐omics sequencing technologies.

**Methods:**

Peripheral blood mononuclear cells (PBMCs) from patients in the acute phase of anti‐NMDARE and healthy controls were sequenced using single‐cell joint profiling of transcriptome and chromatin accessibility. Differential gene expression analysis, transcription factor activity profiling, and cell‐cell communication modeling were performed to elucidate the immune mechanisms underlying the disease. In parallel, single‐cell B cell receptor sequencing (scBCR‐seq) and repertoire analysis were conducted to assess antigen‐driven clonal expansion within the B cell population.

**Results:**

The study revealed a significant clonal expansion of B cells, particularly plasma cells, in anti‐NMDARE patients. The novel finding of type I interferon (IFN‐I) pathway activation suggests a regulatory mechanism that may drive this expansion and enhance antibody secretion. Additionally, activation of Toll‐like receptor 2 (TLR2) in myeloid cells was noted, which may connect to tumor necrosis factor‐alpha (TNF‐α) secretion. This cytokine may contribute to the activation of B and T cells, thereby perpetuating immune dysregulation.

**Conclusions:**

This study presents a comprehensive single‐cell multi‐omics characterization of immune dysregulation in anti‐NMDARE, highlighting the expansion of B cell and the activation of the IFN‐I and TLR2 pathways. These findings provide deeper insights into the molecular mechanism driving the pathogenesis of anti‐NMDARE and offer promising targets for future therapeutic intervention.

**Key points:**

- Significant B cell clonal expansion, particularly in plasma cells, driven by antigen recognition.
- IFN‐I pathway activation in plasma cells boosts their antibody production and potentially exacerbates immune dysregulation.
- TLR2 pathway activation in myeloid cells contributes to TNF‐α secretion and could influence adaptive immune responses.

## Graphical abstract

- Single‐cell multi‐omics analysis revealed distinct immune cell populations and their functional states in anti‐NMDARE.
- Systematical analysis identified key immunoregulation programs involved in disease progression.
- Interactions via cytokines among immune cells may intensify the inflammatory response and disease activity, providing therapeutic implications.

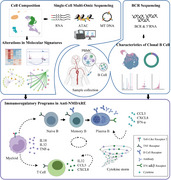

## 1 INTRODUCTION

Anti‐*N*‐methyl‐D‐aspartate receptor encephalitis (anti‐NMDARE) is a serious and rare autoimmune condition distinguished by progressive psychosis, seizures and dysfunction of the autonomic nervous system.^1^, ^2^ It is the most prevalent form of autoimmune encephalitis, representing nearly 81% of all instances, and it affects roughly 1.5 individuals per million annually.^3^ Although known triggers such as tumours and herpes simplex virus are associated with the condition, its causative factors remain largely unidentified.^4^ The pathophysiology of anti‐NMDARE involves the production of IgG antibodies that target the GluN1 component of the NMDA receptor. These antibodies accumulate in the hippocampus, initiating a cascade of neuroinflammatory responses that lead to disruptions to brain function.^2^, ^5^ Current clinical treatments, including B‐cell inhibitors, tumour resection and adjunctive therapies, have been shown to facilitate gradual recovery in only a subset of patients.^1^, ^6^, ^7^, ^8^ Therefore, a deeper understanding of the mechanisms underlying immune dysregulation in this disease is essential for discovering new therapeutic targets and creating more improved treatment strategies.

Single‐cell RNA sequencing (scRNA‐seq) has been utilised to investigate the pathophysiology of anti‐NMDARE. Studies on B cells have revealed an increased proportion of B cells—particularly plasma B cells—in the peripheral blood mononuclear cells (PBMCs) and cerebrospinal fluid of anti‐NMDARE patients.^9^, ^10^ Additionally, elevated levels of B‐cell‐associated cytokines such as CXCL13 and CXCL10 have been observed in the cerebrospinal fluid,^11^, ^12^ while B‐cell receptor (BCR) analysis has also revealed increased levels of NR1‐positive B cells (CD19^+^NR1^+^) in the PBMCs of anti‐NMDARE patients.^13^ These results confirm the critical pathogenic involvement of B cells in this condition. However, the precise mechanisms by which these B cells are activated remain unclear.

Furthermore, the generation of autoantibodies from B plasma cells involves complex mechanisms, including interactions with other immune cells.^14^ Researchers have shown that patients have increased levels of cytokines specific to T cells in their cerebrospinal fluid,^11^, ^12^ as well as increased monocyte and dendritic cell counts in PBMCs.^9^ Although research on myeloid cells in anti‐NMDARE is limited, numerous studies suggest that defects in the innate immune system can trigger B‐cell autoimmunity, potentially mediated by the recognition of viral genomic material and subsequent type I interferon (IFN‐I) production.^15^, ^16^, ^17^, ^18^ These findings indicate that interactions among various immune cells also contribute to the pathogenesis of anti‐NMDARE. However, the precise mechanisms by which these immune interactions are orchestrated remain unclear.

In this study, we sought to elucidate the intricate dynamics governing the activation mechanisms of B cells within the context of anti‐NMDARE pathology. By employing cutting‐edge techniques in single‐cell joint profiling of the transcriptome and chromatin accessibility, as well as single‐cell BCR sequencing, we aimed to comprehensively dissect the regulatory networks and key factors orchestrating the inflammation response of disease‐causing or effective B‐cell populations. Through the integration of multi‐omics and systematic analysis, we aspired to unravel the molecular signatures that exhibit the interactions of immune cells and their regulatory programs in anti‐NMDARE patients at unprecedented resolution, shedding light on novel therapeutic targets for intervention.

## 2 RESULTS

### 2.1 Over‐representation of B cells and monocytes in PBMCs of anti‐NMDARE patients

**FIGURE 1:**
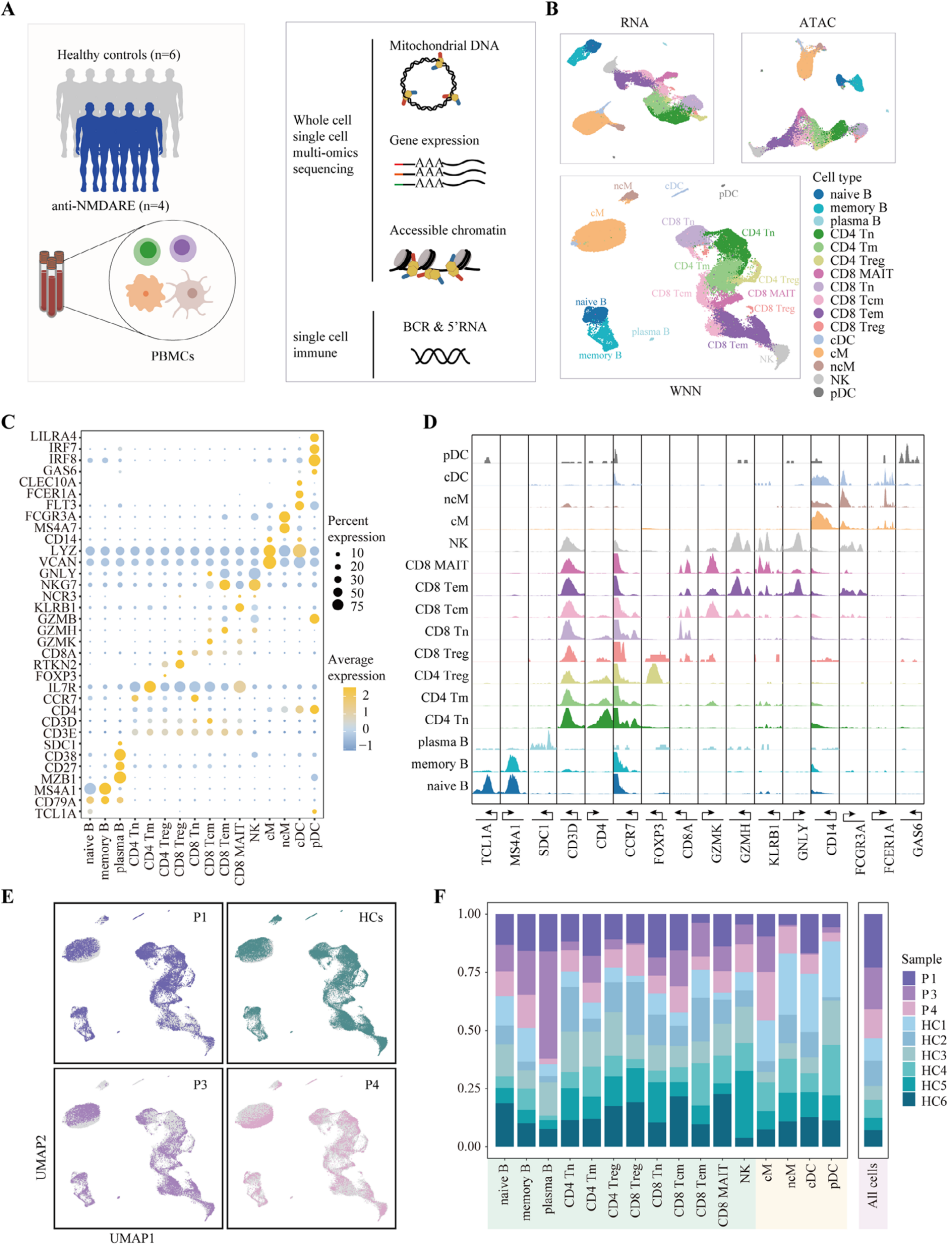
Single‐cell multi‐omics profiling reveals altered immune cell proportions in anti‐NMDARE patients. (A) A schematic representation of the samples and profiling methods used in this study. (B) UMAPs visualising all cells passing quality control, coloured by annotated clusters. Cell annotations are derived from weighted‐nearest neighbour (WNN) assignment of 54 365 cells to 16 cell types: classical and non‐classical monocytes (cMs and ncMs); conventional and plasmacytoid dendritic cells (cDCs and pDCs); naive and memory CD4^+^ T cells (CD4 Tn and CD4 Tm); naive, central memory, effector memory and mucosa‐associated invariant CD8^+^ T cells (CD8 Tn, CD8 Tcm and CD8 Tem, CD8MAIT); regulatory CD4^+^ and CD8^+^ T cells (CD4 Treg and CD8 Treg); nature killer cells (NK); and naive, memory and plasma B cells. (C) A bubble plot displaying the expression of marker genes across each cell type. The size of the dot indicates the proportion of cells in that cell type expressing marker genes. The scaled mean expression of marker genes is represented from light blue to dark blue. (D) Genome tracks illustrating the normalised chromatin accessibility surrounding marker genes. (E) UMAP of WNN graph for single‐cell multi‐omics modalities showing cells per patient and all healthy controls. (F) Proportions of each sample comprising each cell type. Samples from patients with anti‐NMDARE are in purple and healthy controls are shown in green. The total proportions for each sample are shown in the rightmost column.

To elucidate the molecular regulatory principles governing immune cell dynamics in patients with anti‐NMDARE, we conducted high‐throughput single‐cell multi‐omics profiling on PBMCs obtained from four anti‐NMDARE patients at the acute stage before any treatments and six age‐matched healthy donors (Table 1 and Table S1). We optimised the single‐cell multi‐omics protocol using the 10X Chromium platform to simultaneously measure gene expression, chromatin accessibility and mitochondrial genomes from the same cell^19^ (Figure 1A). To reduce library preparation costs, PBMCs from two to four individuals were pooled and sequenced in one library, which was subsequently demultiplexed using MitoSort, a software application we developed to segregate cells based on their germline mitochondrial variants.^20^ Following quantity control of our library and sequencing data (see Method section), we acquired 54 365 cells across five joint‐profiling libraries (Figure S1A,B), which were classified into 16 distinct immune cell populations (Figure 1B) based on the expression patterns and chromatin accessibility of markers unique to each cell type (Figure 1C,D). Patient 2, who yielded insufficient cells, was excluded from downstream analysis. Initial comparisons of cell compositions revealed an over‐representation of plasma B, memory B cells and classical monocytes in anti‐NMDARE patients compared with the healthy controls (Figure 1E,F and Figure S1C). Notably, this observed increase in plasma B cells and monocytes aligns with findings reported by Jiang et al., who studied PBMCs from 10 anti‐NMDARE patients.^9^ Furthermore, the increase in memory B cells aligns with findings from Li et al., who used flow cytometry analysis on PBMCs from 20 acute‐phase anti‐NMDARE patients.^10^ In brief, we presented a detailed, high‐quality, single‐cell, joint profiling map with population census, revealing significant alterations in the cellular compositions of PBMCs in individuals with anti‐NMDARE. These findings underscore these specific cell types' potential roles in the disease's pathology.

**TABLE 1:** Clinical features of the anti‐NMDARE patients studied.

Sample ID	P1	P2	P3	P4
Gender	Female	Male	Male	Female
Age	24	23	18	18
BMI	27.06	18	23.26	18.52
Occupation	Farmer	Undergraduate	Worker	Unemployed
MRS	5	5	5	4
Primary antibody (NMDAR)	+	+	+	+
Initial episode	+	+	+	+
Symptomatology	PD, Sz, RC, MD	PD, Sz, RC, MD	PD, Sz, RC, MD	PD, Sz, MD
Serum antibody	CMV‐IgG+, EBV‐IgG+, EBNA‐IgG+	HSV1‐IgG+, RUBE‐IgG+, CMV‐IgG+	HSV1/2‐IgG+, RUBE‐IgG+, CMV‐IgG+, RUBE‐IgG+	EBV‐IgG+, EBNA‐IgG+
Tumour	–	–	–	PCO

Abbreviations: BMI, body mass index; CMV, *Cytomegalovirus*; EBNA, Epstein–Barr nuclear antigen; EBV, Epstein–Barr virus; HSV1/2, herpes simplex virus type 1/2; MD, movement disorder; MRS, modified rankin scale; NMDAR, *N*‐methyl‐D‐aspartate receptor; PCO, polycystic ovary; PD, psychiatric disorder; RC, reduced consciousness; RUBE, rubella virus; Sz, seizure.

### 2.2 Type I interferon pathway activation in plasma B cells associated with anti‐NMDARE

**FIGURE 2:**
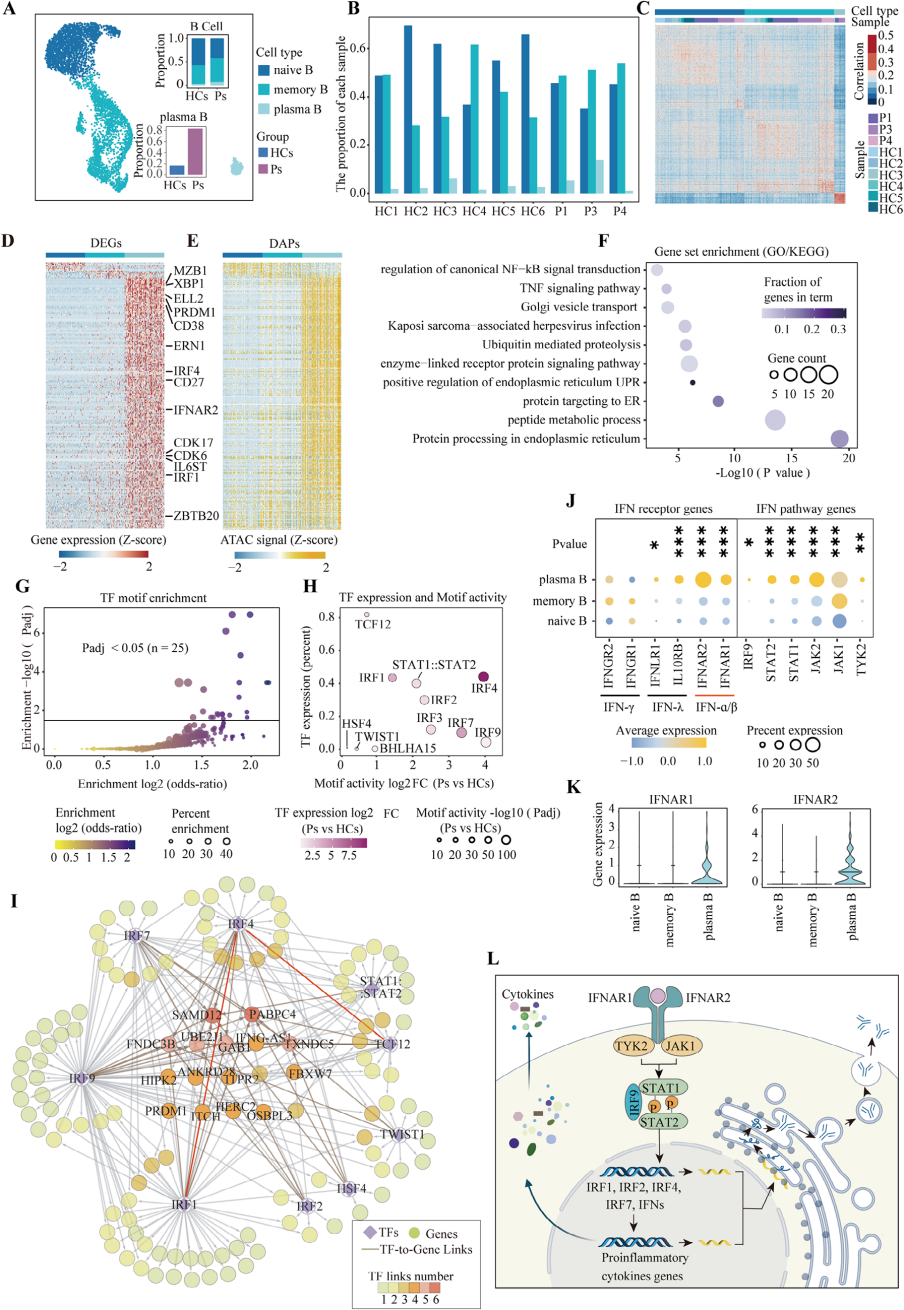
Regulatory map of anti‐NMDARE‐enriched plasma B cells. (A) A UMAP plot displaying all B cells, with identified naive B, memory B and plasma B cells labelled in different colours. Inset: (Top) The stacked bar plots depict the percentages of naive B, memory B and plasma B cells in patients (Ps) and healthy controls (HCs). (Bottom) The stacked bar plots display the percentages of plasma B in Ps and HCs. (B) A bar plot illustrating the percentages of naive B, memory B and plasma B cells in each sample. (C) The Pearson correlation coefficient of each cell type by transcriptome and accessibility matrix. (D) Heatmaps showing expression of differentially expressed genes (DEGs) (Logistic Regression (LR) test, *Padj* ≤ .01, |log2FC| ≥ .5) in 100 cells sampling from naive B, memory B and plasma B, respectively. Each row represents a DEG, and each column represents a sampled cell. The colour indicates the *z*‐transformed gene expression (*z*‐score range is −2 to 2). (E) Heatmap showing differentially accessible peaks (DAPs) in 100 cells sampling from naive B, memory B and plasma B, respectively. Each row represents a DAP, and each column represents a sampled cell. The colour indicates the *z*‐transformed peaks (*z*‐score range is −2 to 2). (F) The Dot plot shows the function enrichment results, the bubble size correlates with the gene count in each term, while the colour represents the percentage of differential genes within each term. (G) A bubble plot visualises all the TFs based on their characteristics. The *Y*‐axis represents the enrichment −log10 (*Padj*), while the *X*‐axis depicts the enrichment score (higher values indicate a stronger match). The size of the bubble represents the enrich percentage, the colour represents the enrichment score of TFs. (H) A bubble plot visualising the expression of the 25 candidate TFs (enrichment *Padj *< .05). The size represents the −log10 (*Padj*), and the colour represents the log2FC of these TFs expressions. (I) A gene regulation network (GRN) depicting the key TFs and the genes regulated. (J) A Dot plot depicting the gene expression level of the interferon (IFN) pathway in B cells. Dot size correlates with the percentage of cells within each cluster, while the colour represents the average expression level. The *p*‐values were calculated by the one‐way ANOVA method, *p < .05, **p < .01, ***p < .001. (K) The violin plot depicts the expression level of *IFNAR1* and *IFNAR2* receptor genes in naive B, memory B and plasma B cells. (L) A schematic illustrating activation of the type I interferon (IFN‐I) pathway in plasma B cells.

The plasma B cells derived from activated B cells have been established as critical disease‐causal cells in anti‐NMDARE.^6^ Notably, the plasmablasts directly produce NMDAR autoantibodies and are continuously replenished by the ongoing differentiation of memory B cells.^9^, ^21^, ^22^, ^23^ Although the functional roles and treatment targets of these cells are well‐documented, the underlying activation and perpetuation mechanisms are incompletely understood. To address this knowledge gap, we focused on the B‐cell populations (*n* = 4963) to delineate the detailed characteristics of naive B, memory B and plasma B cells in anti‐NMDARE (Figure 2A,B and Figure S2A). Upon comparing both transcriptome and chromatin profiles, we observed only mild differences between naive and memory B cells from patients and healthy controls (Figure 2C–E). These results suggest that a limited subset of cells within the naive or memory B‐cell population contribute to the disease, aligning with the principle of heterogeneous responses in adaptive immune cells. Given that plasma B cells are almost patient‐specific (Figure 2A), we conducted an in‐depth molecular regulatory analysis of plasma B cells compared with other B‐cell types to investigate the regulatory programs underlying their activation and functions in anti‐NMDARE. Differential analysis identified a set of differentially expressed genes (DEGs) and accessible peaks (DAPs), corresponding to cis‐regulatory elements (CREs) in plasma B cells (Figure 2D–E). These DEGs were enriched for transcription factors (TFs) associated with plasma B activation, such as *IRF4* (interferon regulatory factor 4), *IRF1* (interferon regulatory factor 1), *PRDM1* (encoding PR/SET domain 1) and *XBP1* (X‐box binding protein 1),^24^, ^25^ as well as genes crucial for immunoglobulin (IG) assembly and secretion, including *ELL2* (elongation factor for RNA polymerase II), *ZBTB20* (zinc finger and BTB domain containing 20), *ERN1* (encoding Inositol‐requiring enzyme‐1) and *HSPA5* (heat shock protein family A member 5)^26^, ^27^, ^28^ (Figure 2D). Functional analysis of these upregulated genes reveals that plasma B cells demonstrate an enhanced expression of genes pertinent to the protein processing machinery within the endoplasmic reticulum (ER). These genes are significantly associated with unfolded protein response (UPR) and Golgi vesicle transport, as is consistent with their cellular role in antibody secretion. Furthermore, these upregulated genes involved in the signalling pathways of tumour necrosis factor (TNF) and nuclear factor kappa B (NF‐κB) indicate a role of plasma B in the production of proinflammatory cytokines (Figure 2F and Table S2).^29^ These findings underscore the molecular landscape governing plasma B‐cell function in anti‐NMDARE.

To clarify the regulatory mechanisms of these genes involved in plasma cell functions, we performed motif enrichment analysis on the accessible chromatin regions of genes significantly correlated with their expression. By analysing the TF binding motifs enrichment (*Padj* < .05, Figure 2G) and the expression patterns of these enriched TFs (Figure 2H), we identified the interferon regulatory factor (IRF) family, particularly *IRF4*, *IRF1* and *IRF2*, as the most prominent regulators. Additionally, our analysis highlighted other important TFs, including *STAT* and *TCF12*, which also play roles in modulating plasma B‐cell functions (Figure S3A–C). Using binding scores in the peak sets near DEGs for these key TFs, we constructed a TF‐gene regulation network (GRN) to elucidate the complex regulatory programs modulating the transcriptome dynamics of plasma B cells (Figure 2I and Table S3). We identified the IRF family and STAT families as key components within the network. Specifically, *STAT1* and *STAT2*, along with *IRF9*, are integral to the IFN system, while *IRF1*, *IRF2*, *IRF7* and *IRF4* have been demonstrated to be upregulated by IFN stimulation.^30^, ^31^, ^32^, ^33^, ^34^, ^35^, ^36^ Our GRN revealed that *IRF1* and *TCF12* govern *IRF4* through distinct enhancers (Figure 2I and Figure S3D). This indicates a potential cascade or synergistic effect among these TFs, exerting multifaceted regulatory control over the functional characteristics of plasma B cells.

In addition, we observed a significant upregulation of genes encoding IFN‐I receptor proteins, specifically IFNAR1 and IFNAR2 in plasma B cells of anti‐NMDARE (Figure 2J,K). These observations suggest that the IFN‐I receptor pathway functions as the upstream signal, modulating these transcriptional regulators and their downstream targets, thereby boosting antibody secretion and triggering pro‐inflammatory cytokines production (Figure 2L). These findings offer a detailed perspective on the complex regulatory networks in plasma B cells, providing insights into the transcriptional control mechanisms that underpin their role in anti‐NMDARE. Understanding these regulatory interactions is crucial for developing targeted therapeutic strategies aimed at modulating plasma B‐cell activity in disease contexts.

### 2.3 Type I interferon pathway activation in B cells undergoing clonal expansion targeting NMDAR

**FIGURE 3:**
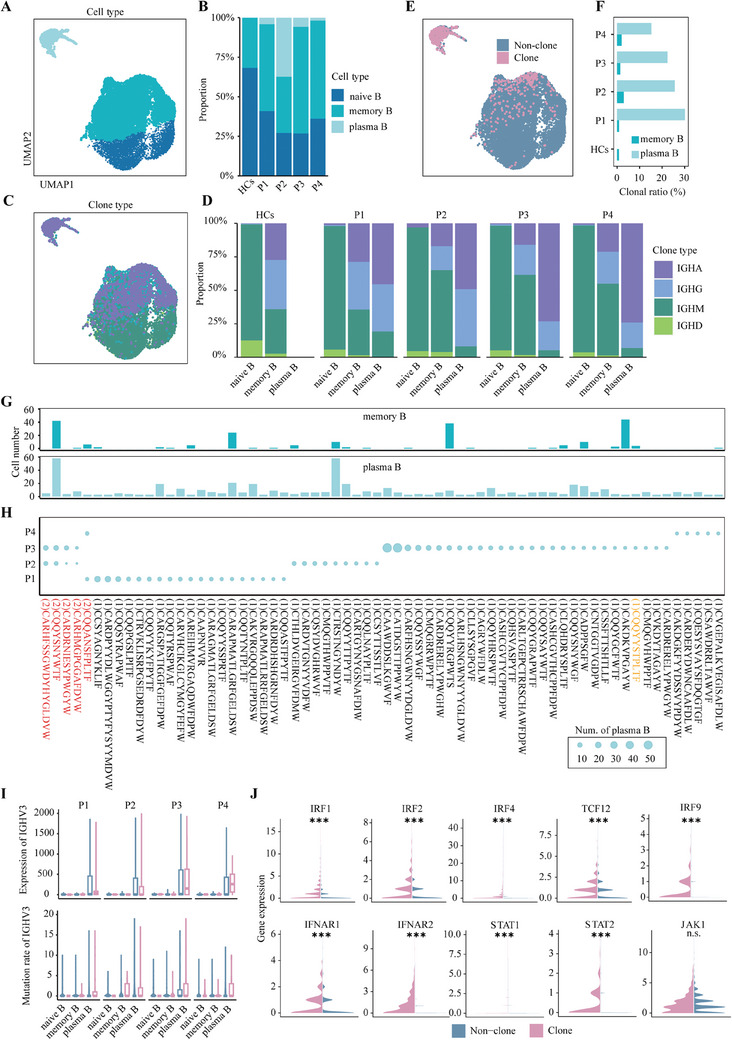
B‐cell immune activation in anti‐NMDARE. (A and B) A UMAP plot displaying naive B, memory B and plasma B cells labelled in different colours (A). The stacked bar plots depict the proportion of naive B, memory B and plasma B cells in each Ps and HCs (B). (C and D) A UMAP plot displaying four clone types in B cells (C). The bar plot indicates the proportion of four clone types in naive B, memory B and plasma B cells (D). (E and F) A UMAP plot displaying clones and non‐clones in B cells (E). The proportion of clones in plasma B and memory B cells across individual samples (F). (G and H) Clonal types were identified within each sample. The bar plot (G) illustrates the cell count of memory B and plasma B cells within each clonal type. The dot plot (H) represents the number of plasma B cells within clonal types across samples. The numbers on the horizontal axis labels denote the count of each clonal type in Ps. (I) Expression levels and mutation rates of the *IGHV3* gene in naive B, memory B and plasma B cells, with colours indicating whether the cells are clonal or not. (J) Expression levels of genes in the IFN‐I pathway across B cells, with colours indicating whether the cells are clonal or not (Wilcoxon rank sum test). *p < .05, **p < .01, ***p < .001.

B‐cell activation by specific antigens, recognised through their cell‐specific receptors, triggers clonal expansion and differentiation processes from naive or memory B cells to plasma B cells. To elucidate how the IFN‐I pathway is involved or coordinated with activation by BCRs, we further conducted single‐cell 5′ RNA and BCR sequencing on B cells. B cells enriched from first‐episode anti‐NMDAR patients were pooled to construct a single‐cell BCR immune sequencing library. In the same way, germline variants were used to demultiplex cells into different individuals^37^ (Figure S4C,D, Methods). The cell types were annotated using marker genes (Figure 3A and Figure S4A,B). A comparative analysis of BCR sequencing data from anti‐NMDARE patients against a public database of healthy controls^38^ revealed a notable increase in plasma B and memory B cells in the patients (Figure 3B).

The antibody classes of B cells are defined by the constant regions of their IG‐heavy chains.^39^ To differentiate the antibody types of B cells, we characterised IG gene expression patterns in B cells from anti‐NMDARE patients (Figure 3C,D). We observed that non‐clonal naive B cells predominantly express *IGHM* and *IGHD*. In comparison, the memory B‐cell subset demonstrates a marked increase in *IGHG* and *IGHA* expression, suggesting their engagement in a wider array of antigen‐recognition processes (Figure 3D).^40^ In differentiated plasma cells from anti‐NMDARE patients, *IGHA* expression is higher compared to that for memory B cells, while *IGHD* and *IGHM* expression is significantly reduced or absent. This indicates that the body has been exposed to a variety of viral, bacterial or protein antigens.^39^ In addition, through an in‐depth examination of BCR sequences, particularly the complementarity‐determining regions (CDR) of the immunoglobulin heavy chain (IGHV), we identified a significant clonal expansion of plasma B cells, whereas a less pronounced clonal population within memory B cells (Figure 3E,F and Figure S4F). Clonal sharing was observed among several subsets within the patient cohort. A clone distinguished by the CDR sequence CQQYYYSTPLTF, discovered in Patient 3, matched a clone previously reported in anti‐NMDARE research^10^ (Figure 3G,H). This suggests that the antigen causing clonal expansion may be the common NMDAR shared among the patients. The IGHV mutations in B cells arising in the germinal centres, known as somatic hypermutation (SHM), particularly *IGHV3*, are a hallmark of antibody diversity and a manifestation of B‐cell activation.^41^ A significant upregulation of the *IGHV3* gene was identified in these clonal B cells, accompanied by a substantial number of somatic mutations^42^ (Figure 3I and Figure S4G). Concurrently, these clonal B cells demonstrate an increase in genes associated with the IFN‐I signalling pathway and the aforementioned sequential TFs (Figure 3J).

Overall, these findings suggest that in anti‐NMDARE patients, B cells differentiate and expand into memory B and plasma B cells through specific antigen recognition, associated with SHM in the *IGHV3* region of the BCR heavy chain. The upregulation of the IFN‐I pathway and its major downstream TFs in these expanded B cells enhances antibody secretion and inflammatory cytokine production, underscoring the potential role of IFN‐I in anti‐NMDARE pathogenesis.

### 2.4 T‐cell dysregulation in anti‐NMDARE enriched with NF‐κB and cytokine signalling pathways

**FIGURE 4:**
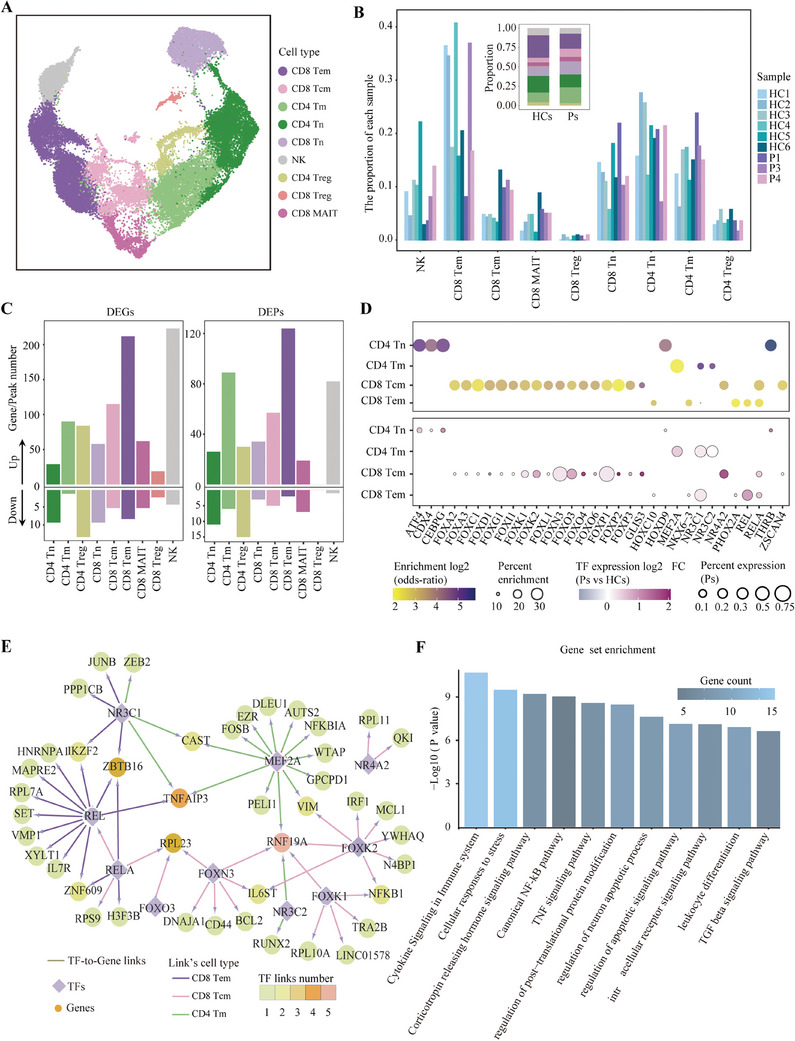
Dysregulation of T cells in anti‐NMDARE. (A) A UMAP plot displaying all T and NK cells, coloured by annotated clusters. (B) The proportion of T and NK cells in each sample. Inset: The proportion of T and NK cells in Ps and HCs (Likelihood Ratio test, *Padj* ≤ .01, |log2FC| ≥ .5). (C) A bar plot displaying the number of DEGs (left) and DAPs (right) in Ps compared with HCs. (D) A bubble plot displaying the enrichment score of motifs (top) and the expression and chromVAR activity of TFs (bottom). (E) The regulation network (TF‐gene) in CD4 Tm, CD8 Tcm and CD8 Tem. The colour of nodes represents the number of edges, and the colour of edges indicates the T‐cell subtype. (F) A bar plot depicting the function enrichment results of genes in (E).

B cells, upon antigenic stimulation, undergo a process of activation often accompanied by the activation of T cells.^43^ Given that activated T cells can circulate systemically, it is plausible that T cells within the PBMCs might exhibit distinct molecular signatures relevant to anti‐NMDARE. We compared T‐cell subsets between anti‐NMDARE patients and healthy controls, and found no significant variations in the composition of T‐cell subsets (Figure 4A,B and Figure S2B). However, we observed a notable prevalence of upregulated DEGs and open chromatin regions across nearly all T‐cell subtypes, with upregulated DEGs outnumbering downregulated ones (Figure 4C and Figure S5A,B).

To identify the regulatory factors responsible for the activation in T‐cell subtypes, we conducted motifs enrichment analysis on the open chromatin regions that were upregulated in each T‐cell subtype. This analysis revealed enriched regulatory motif patterns across four subtypes: naive CD4^+^, memory CD4^+^, central memory CD8^+^ and effector memory CD8^+^ T cells (Figure S5C). We selected motifs (*p*‐value <.05 and fold enrichment score >2) and characterised the expression levels of their corresponding TFs in anti‐NMDARE patients and healthy controls (log2FC > 0 and an expression ratio in patients >.05) (Figure 4D). This approach identified several key TFs within three memory T‐cell subtypes, including components of the NF‐κB complex such as *REL* and *RELA*, glucocorticoid receptors *NR3C1* and *NR3C2*, and select members of the *FOX* family. By utilising the binding affinities of these pivotal TFs to the peak sets flanking DEGs, we delineated a TF‐gene regulatory network unique to these three memory T‐cell subtypes (Figure 4E and Table S4). Functional analysis of this regulatory network, which contributes to T‐cell activation in anti‐NMDARE, revealed significant enrichment in functions associated with immune response and regulation, including cytokine signalling pathways within the immune system and the TNF and canonical NF‐κB pathways (Figure 4F and Table S5).

Collectively, these observations imply that peripheral T cells in anti‐NMDARE patients may engage in diverse immunological activation pathways mediated by critical TF‐driven gene cascades. This is particularly evident in memory T cells, where activation pathways may facilitate the production of pro‐inflammatory cytokines, thereby exacerbating the inflammatory response and contributing to immune dysregulation in anti‐NMDARE.

### 2.5 Myeloid cell‐mediated interactions in the pathogenesis of anti‐NMDARE

**FIGURE 5:**
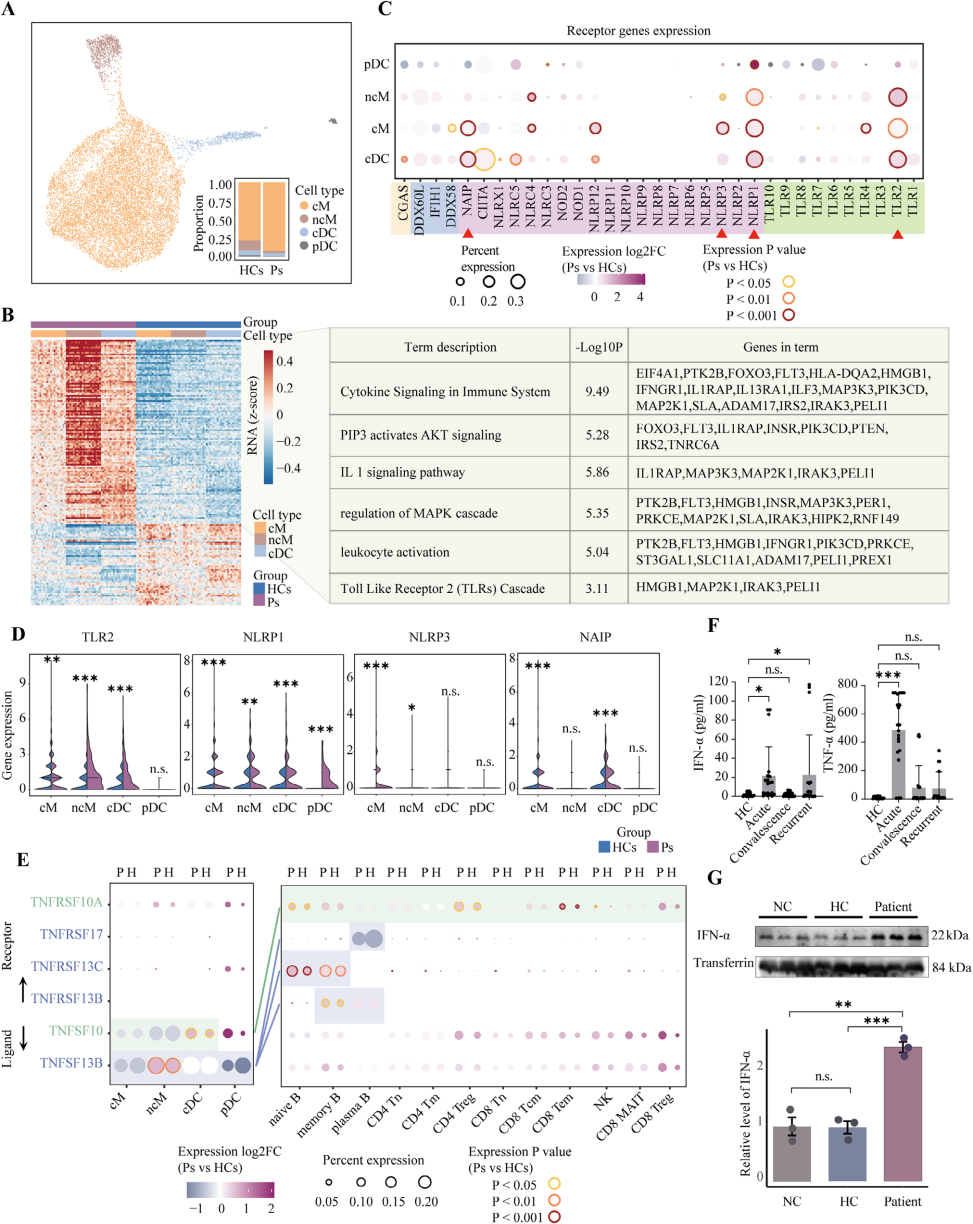
Dysregulation of myeloid cells in anti‐NMDARE. (A) A UMAP plot displaying four myeloid cell subtypes, coloured by annotated clusters. Inset: The proportions of four types of myeloid cells in Ps and HCs. (B) Left: A heatmap showing the expression of DEGs (Likelihood Ratio test, *Padj* < .01, |log2FC| > .5) in 100 cells sampling from three myeloid cell subtypes, respectively. Each row represents a DEG, and each column represents a sampled cell. The colour indicates the *z*‐transformed gene expression (*z*‐score range is −.5 to .5). Right: Function enrichment results of the upregulated genes in cMs, ncMs and cDCs, respectively. (C) A bubble plot displaying the antigen receptor protein‐encoding gene expression levels in four myeloid subtypes. The colour represents the expression ratio, and the size represents the log2FC between Ps and HCs. The border colours of the dots indicate the significance (*p*‐value by Wilcox rank sum test) of the comparison. The red triangle marks the four most significantly different genes. (D) A violin plot depicting the expression levels of four receptor genes in four myeloid subtypes in Ps and HCs (Wilcoxon rank sum test). *p < .05, **p < .01, ***p < .001. (E) A bubble plot depicting the expression levels of TNF pathway ligand–receptor genes in 16 immune cell subtypes. The colour of the bubbles represents the log2 fold‐change in expression between Ps and HCs, while the border colour indicates the *p*‐value of the difference (Wilcoxon rank sum test). The size of each bubble corresponds to the percentage of expression. The background shading highlights significant cell–cell interactions by CellChat (*p* < .01), and the different background frames and central lines represent distinct ligand–receptor pairs. (F) The levels of IFN‐α and TNF‐α in plasma of HCs (*n* = 7), acute phase anti‐NMDARE (*n* = 7), convalescence anti‐NMDARE (*n* = 7) and recurrent stage anti‐NMDARE (*n* = 6). Each sample was processed in three technical replicates. The *p*‐values were calculated by the one‐way ANOVA method, *p < .05, **p < .01, ***p < .001. (G) The levels of IFN‐α in the serum of humanised mice engrafted with PBMCs from anti‐NMDARE patients (Patient), healthy donors (HC) and medium‐only controls (NC) were assessed using Western blot analysis. Each group comprised three samples. *p*‐Values were calculated using *T*‐test, with significance defined as *p < .05, **p < .01 and ***p < .001.

The innate immune system, acting as a critical immunological checkpoint, is invariably integral to the immune response. Emerging research indicates that innate immune cells dictate the activation of adaptive immune cells in B‐cell systemic autoimmunity.^14^ Consequently, we delineated the contributions and signatures of the innate immune system within anti‐NMDARE, noting a pronounced prevalence of monocytes, which is the predominant cell subset within the innate immune repertoire (Figure 5A and Figure S6A).

By comparing the DEGs within each myeloid subtype (excluding plasmacytoid dendritic cells [pDCs] due to their low numbers) between anti‐NMDARE patients and healthy controls (Figure 5B and Figure S6B), we identified a total of 107 upregulated DEGs. Interestingly, we found that these upregulated genes are involved in the Toll‐like receptor 2 (TLR2) cascade and its downstream pathways, including the PIP3‐mediated activation of the AKT signalling pathway, IL‐1 signalling and the MAPK cascade.^44^ These findings strongly suggest a propensity of myeloid cells to initiate a cascade of responses via TLR receptors, indicating a potential role in the immune response observed in anti‐NMDARE patients.

In addition to TLRs, other membrane receptors are capable of activating myeloid cells, including cyclic GMP‐AMP synthase (cGAS),^45^ RIG‐I‐like receptors (RLRs)^46^ and NOD‐like receptors (NLRs).^47^, ^48^ To achieve a more comprehensive understanding of the activation pathways in myeloid cells, we quantified the differential expression levels of genes encoding these receptor proteins in anti‐NMDARE patients versus healthy controls (Figure 5C,D). Our analysis revealed significant upregulation of the transmembrane receptor TLR2 and the cytosolic inflammasome NLRP1 in anti‐NMDARE, indicating their possible involvement in the functional execution of myeloid cells in anti‐NMDARE. This indicates that these receptors could be essential in the pathophysiological processes associated with the disease. Verification of the expression levels of key downstream genes of TLR2 further revealed that the activation of TLR2 induces a series of downstream cascade reactions, encompassing the canonical MAPK and NF‐κB pathways (Figure S6F). Studies have shown that cytosolic inflammasomes, with the assistance of TLR downstream factors, synthesise and contribute to the maturation and secretion of cytokines, thereby facilitating the pro‐inflammatory functions of the TLR pathway.^49^, ^50^ These findings suggest that the combined action of TLR2 and NLRP1 triggers the secretion of inflammatory cytokines, such as interleukins, chemokines and TNF, which also act as mediators for the interaction between myeloid cells and other immune cells.

We further investigated the dynamic interplay between myeloid cells and adaptive immune cells, as orchestrated by extracellular secretion of cytokines.^51^ Our findings underscore a pronounced increase in the intercellular interactions and their involved pathways in patients with anti‐NMDARE, particularly those involving several subtypes of myeloid cells that exhibit greater changes and higher levels of involvement (Figure S6G,H). Subsequent analysis of the expression levels of ligand–receptor pairs in these pathways revealed TNF pathway genes exhibiting the most significant differential expression (Figure 5E). Specifically, the ligand gene *TNFSF13B*, prominently expressed in myeloid cells, was dramatically upregulated in the non‐classical monocytes (ncMs) of anti‐NMDARE patients. Correspondingly, its receptor genes *TNFRSF13B* and *TNFRSF13C* displayed significant upregulation in B cells. These observations suggest that ncMs, possibly activated via the interaction between TLR2 and NLRP1‐mediated priming, secrete TNF to instigate the TNF pathway in B cells. Moreover, another ligand gene in the TNF pathway, *TNFSF10*, demonstrated significant upregulation in circulating dendritic cells (cDCs) in the patients. Concurrently, the corresponding receptor gene *TNFRSF10A* exhibited upregulation in the majority of lymphoid cells, indicating a broad myeloid cell‐mediated activation of the adaptive immune response through an alternative TNF factor. This activation is exemplified by the observed activation of TNF and its downstream NF‐κB pathway in T cells.

To confirm that transcriptome‐level dysregulation extends to the functional level, we further performed a quantitative analysis of TNF‐α and IFN‐α protein levels in plasma (Figure 5F). The results revealed a notable elevation in the levels of both cytokines during the acute phase among anti‐NMDARE patients, surpassing those of the other groups. Notably, a pronounced elevation of IFN‐α was also detected in the relapse group. Additionally, we measured IFN‐α levels in the serum of our previously established humanised anti‐NMDARE mouse model,^52^ and found a significant elevation compared to the control groups (Figure 5G and Figure S7). This result from the well‐controlled mouse model rules out other uncontrolled variables that may contribute to the differences observed in clinical samples, underscoring the involvement of IFN‐α and TNF‐α in the pathophysiology of anti‐NMDARE.

In summary, our analysis delineates a putative immunoregulatory pattern observed in patients with anti‐NMDARE (Figure S8). Myeloid cells, upon activation via TLR2 and assisted by inflammasome NLRP1, trigger a cascade of events that promote the nuclear translocation and maturation of inflammatory cytokines, notably TNF, which are subsequently secreted. Distinct TNF molecules then bind to TNF receptor proteins on the surfaces of B and T cells, thereby activating the TNF signalling pathway. This activation further facilitates the maturation and differentiation of B cells and also initiates downstream immune stress pathways, such as NF‐κB, in T cells. Concurrently, these inflammatory factors can aid B plasma cells in engaging the IFN‐I pathway, thereby promoting antibody secretion.^53^ Ultimately, the activation of these cells may enhance the release of autoinflammatory cytokines, including TNF‐α and IFN‐α, and amplify intercellular interactive responses. This multi‐cellular positive feedback regulatory network with the myeloid as a key point may be one of the significant causes of acute onset in patients. It also underscores the pivotal role of myeloid cells in the pathogenesis of autoimmune encephalitis, suggesting that TLR2 could serve as a potential therapeutic target to dampen this positive feedback cascade, thereby mitigating disease progression or aiding the efficacy of other treatment modalities.

## 3 DISCUSSION

In this study, we utilised single‐cell multi‐omics sequencing technology to comprehensively analyse the major regulatory networks and activation pathways of various immune cells in the PBMCs of anti‐NMDARE patients for the first time. Our findings highlight the significant role of myeloid cells in addition to the well‐documented critical involvement of B cells in the disease's pathogenesis.

In agreement with earlier research, we noted increased proportions of plasma cells, memory B cells and monocytes in patients' peripheral blood.^9^, ^10^ Changes in PBMCs reflect alterations in the immune system, underscoring the pivotal role of B cells in this disease and suggesting a potentially important role for monocytes as well.

While the optimisation of antigen specificity through SHM in B cells is well established,^42^ our study further identifies that the genes undergoing high‐frequency mutations in anti‐NMDARE predominantly belong to the *IGHV3* family. This observation is consistent with other autoimmune and infectious diseases,^54^, ^55^ where mutations frequently target the IGHV region, indicating a shared mechanism in B‐cell‐mediated immune responses. Additionally, we found that the activation pathway of these antibody‐secreting plasma cells is the IFN‐I pathway. In the context of IFN‐I pathway activation, key TFs such as IRF and STAT families are upregulated, regulating a set of downstream target genes. These TF‐gene regulation networks collectively govern plasma B‐cell functions, which include antibody secretion, participation in inflammatory responses and modulation of cellular metabolism.^56^ This regulation facilitates the activation and functional adaptation of plasma cells to perform their roles in immunity. Numerous studies have demonstrated that the IFN pathway is essential in autoimmune diseases, particularly in systemic lupus erythematosus and type I diabetes mellitus, by promoting T‐ and B‐cell proliferation and survival, enhancing antibody production and facilitating IG class switching.^14^, ^57^, ^58^, ^59^ Our findings suggest that IFN‐I may similarly contribute significantly to the pathogenesis of anti‐NMDARE.

Research on innate immune cells, particularly myeloid cells, in anti‐NMDARE is very limited. The few studies available have identified certain changes in peripheral myeloid cells without further elucidating their specific roles. Our analysis is the first to suggest that myeloid cells may exert their activation function through the TLR2 receptor, triggering downstream pathways to release inflammatory cytokines, thereby promoting the adaptive immune response. NLRP1 appears to have a supportive function in this process. As a member of the Toll‐like receptor family, TLR2 activates signalling pathways by recognising various pathogen‐associated molecular patterns, which trigger both innate and acquired immune responses.^60^, ^61^ This process is typically mediated by multiple cytokines. In addition to our findings of upregulated gene expression in the TLR2 pathway, elevated levels of cytokines such as IL‐6, CXCL13, TNF‐α and NLRP3 inflammasome components in the cerebrospinal fluid of anti‐NMDARE patients have been reported in some studies, which is consistent with TLR2 downstream activation.^62^, ^63^ These findings indicate that TLR2 may act as a critical trigger, playing a significant role in anti‐NMDARE.

Given the role of these inflammatory cytokines in mediating interactions between immune cells, our analysis elucidates the specific function of TNF‐α in anti‐NMDARE. As a downstream cytokine of TLR2, TNF‐α mediates cell interactions through different ligand–receptor pairs (TNFSF13B–TNFRSF13B/13C/17 and TNFSF10–TNFRSF10A), which interact with B and T cells, respectively. These interactions often lead to a positive feedback loop, where cytokine secretion promotes further immune cell activation, resulting in an amplification of the inflammatory response.^64^, ^65^

In summary, the activation of TLR2 and its downstream pathways in myeloid cells promotes the release of multiple cytokines, particularly TNF‐α. These cytokines interact with adaptive immune cells, including B and T cells, thereby amplifying the immune response. This creates a positive feedback loop of cytokine secretion, further amplifying the inflammatory response and advancing disease progression. Among the activated pathways, in addition to inflammation‐related pathways such as NF‐κB, the IFN‐I pathway is most prominently activated in B cells, playing a crucial role in anti‐NMDARE by promoting antibody secretion (Figures S8 and S9). This is the first proposed interaction map of immune cells in anti‐NMDARE. These findings provide a comprehensive perspective on the pathogenesis of anti‐NMDARE and suggest potential therapeutic targets for clinical research, such as TLR2 on myeloid cells or IFNARs on plasma B cells. Drugs that modulate the TNF‐α and IFN‐I pathways might help reduce the inflammatory cascades that amplify the immune response and could potentially be considered candidates for therapeutic intervention, particularly in combination with current clinical treatment to restore immune balance and prevent relapse.^66^, ^67^ In addition to the potential therapeutic targets identified in our study, these molecular features may also serve as potential biomarkers for monitoring immune balance. Biomarkers such as TNF‐α, and IFN‐α, can be readily assayed through blood tests and might be useful in evaluating the efficacy of therapeutic interventions,^68^, ^69^ allowing for adjustment to treatment plans as needed. The regulatory pathways and cell–cell interactions we have proposed provide a framework that could potentially be translated into clinical practice, facilitating more personalised and effective treatment strategies for patients with anti‐NMDARE.

However, our study has several limitations. The low incidence rate of the disease, combined with our strict criteria, which restricted the cohort to patients experiencing their first episode, the sample size for the multi‐omics analysis was relatively small. As a result, we were unable to gather sufficient cerebrospinal fluid samples to analyse immune cells and capture the inflammatory response comprehensively. Although we controlled for factors such as age and gender, the small sample size prevented us from adequately assessing other variables, such as disease severity and medical history. Furthermore, our study population primarily consisted of Asian patients, which may introduce a population bias and limit the generalisability of our findings to other ethnic groups. Future studies should aim to include a more diverse patient population, as well as patients with varying susceptibility factors, such as herpes simplex encephalitis, teratoma and other unknown triggers to better elucidate the relationship between different predisposing factors and the immunoregulatory programs.

## 4 METHODS AND MATERIALS

### 4.1 Participants

Peripheral blood samples were consecutively collected from four patients (one male and three females) who presented to the Sun Yat‐sen University's Third Affiliated Hospital with their first episode of anti‐NMDARE between July 2021 and June 2022. All patients met the diagnostic criteria for definite anti‐NMDARE as outlined by Graus et al. (2016).^70^ Individual information for each participant is summarised in Table 1. Peripheral blood from six healthy donors (three males and three females) was collected as control samples. The age of the recruited subjects did not show any notable statistical differences (anti‐NMDARE patients vs. healthy controls: 20.75 ± 3.25 vs. 23 ± 1 years, *p* = .257). The criteria for including healthy controls were: (i) being over 18 years old; and (ii) generally in good health, with a body mass index ranging from 18 to 34 and normal results in standard laboratory tests. For patients diagnosed with active anti‐NMDAR, the inclusion criteria were: (I) being over 18 years old; and (II) having a diagnosis of active anti‐NMDAR and no treatment with cortisone or immune‐modulating drugs. Exclusion criteria included acute myocardial infarction, heart failure, liver disease (such as cirrhosis, fatty liver and viral hepatitis), haematological disorders or any haemodynamic instability present at the time of obtaining consent. For the patients and healthy controls, peripheral blood was collected in EDTA tubes and processed within 4 h of collection.

### 4.2 Isolation of PBMCs and B cells

Peripheral blood was diluted in a 1:1.5 ratio with .9% physiological saline. PBMCs were then isolated using Lymphoprep (STEMCELL) density gradient. B cells were extracted from PBMCs using Miltenyi CD19 MicroBeads (Miltenyi Biotec, 130‐050‐301).

### 4.3 Single‐cell multi‐omics sequencing libraries preparation and sequencing

Single‐cell multi‐omics libraries were performed as previously described with some modifications to increase the yield of mitochondrial DNA (mtDNA).^71^ After washing twice with PBS, cells were fixed using 1% formaldehyde (Thermo, 28906) in PBS for 10 min at room temperature (RT). The fixation was subsequently quenched using a glycine solution (Sigma, G7126‐500g) to a final concentration of .125 M, and held on ice for 5 min. Cells were washed twice with chilled buffer (PBS, 1 mM DTT, 1 U/µL Protector RNase Inhibitor) and centrifuged at 400 × *g* for 5 min at 4°C. Subsequently, cells were treated with chilled lysis buffer for 3 min on ice, followed by the addition of 1 mL of chilled wash buffer. The mixture was inverted and centrifuged at 500 × *g* for 5 min at 4°C. Cells were then washed twice with chilled wash buffer. All centrifugations were performed using a swing bucket centrifuge. After the supernatant, cells were suspended in a diluted nuclei buffer (nuclease‐free water, 1× diluted nuclei buffer, 1 mM DTT, 1 U/µL Protector RNase Inhibitor) before counting. Following tagmentation, cells were transferred to a Chromium Controller microfluidics device to create single‐cell gel bead‐in‐emulsions (GEMs), after which linear PCR was performed according to the protocol. After breaking the GEMs, the barcoded tagmented DNA and barcoded RNA were purified and subsequently amplified to facilitate sample indexing and library enrichment. The final libraries were quantified with a Qubit dsDNA HS Assay Kit (Invitrogen, Q33231), and assessed using a high‐sensitivity DNA chip (Agilent, 5067‐4626) on a Bioanalyzer 2100 system. The final libraries were sequenced on the NovaSeq 6000 platform (Illumina) in paired‐end mode.

### 4.4 Single‐cell 5′ RNA and B‐cell receptor libraries preparation and sequencing

B‐cell suspension was processed with the B‐cell Single Cell V(D)J solution before being transferred to the Chromium Controller single‐cell instrument, following the manufacturer's recommendations (10X Genomics). SPRIselect beads were employed to fractionate antigen barcode libraries from cellular mRNA libraries. The resulting libraries were PCR amplified for 12 cycles. Preparation of the cellular mRNA library continued according to the suggested protocols by 10X Genomics, producing libraries compatible with Illumina sequencing. The libraries of single‐cell 5′ RNA and BCRs were sequenced on the NovaSeq 6000 platform (Illumina). Additionally, normal data of single‐cell 5′ RNA and BCRs were obtained from public datasets at GEO (GSE194245).

### 4.5 Cytokine release assay

The plasma was collected using an anticoagulant tube and centrifuged at 3000 × *g* for 10 min at 4°C. Subsequently, plasma was carefully collected, divided into aliquots and stored at −80°C until use. The cytokine levels of plasma were measured using Human Immunoassay Valukine ELISA Kits for IFN‐α (Elabscience Biotechnology, E‐EL‐H6125)^68^ and TNF‐α (Elabscience Biotechnology, E‐EL‐H0109c),^69^ following the manufacturer's instructions. Data were acquired using microplate readers (TECAN infinite M200 Pro Nanoquant).

### 4.6 Western blot

We used a humanised mouse model of anti‐NMDARE as previously described by intraperitoneal injection of patients’ PBMCs into BALB/c Rag2^−/−^Il2rg^−/−^SirpαNODFlk2^−/−^ mice.^52^ The mice were injected with cell medium as negative control (NC, *n* = 3). The mice were injected with PBMCs from healthy individuals as healthy control (HC, *n* = 3), and mice were injected with PBMCs from anti‐NMDARE patients as the patient group (Patient, *n* = 3). The venous blood of the mice was collected, and immediately after collection, serum was isolated by centrifugation at 3000 × g for 10 min at 4°C. The protein concentration of serum from NC, HC and patient groups was determined using the Pierce BCA Protein Assay Kit (ThermoFisher, A55865). The serum samples were separated on a 12% SDS‐PAGE gel and transferred onto PVDF Western blotting membranes (Roche, 3010040001). The membranes were blocked with 5% skimmed milk for 3 h. Next, the membranes were incubated with anti‐IFN‐α (Affinity, #DF6086, 1:1000) or anti‐transferrin (Abcam, ab84036, 1:1000) primary antibodies overnight at 4°C, respectively. Transferrin was used as an internal control. After twice washing with Tris buffered saline with tween 20 (TBST) for 10 min at RT, the membranes were incubated with Goat anti‐Rabbit IgG (H+L) (Invitrogen, #31460, 1:100 000) secondary antibody for 3 h at RT. Then, the signals were detected using an enhanced SuperKine kit (Abbkine) and visualised in the Tanon‐5200CE system. Finally, the optical density of each band was quantified using ImageJ.

### 4.7 Preprocessing of single‐cell 5′ RNA and BCR sequencing data

The obtained single‐cell 5′ RNA and BCR sequencing data were cleaned and aligned using Cell Ranger (version 7.1.0) with the GRCh38 genome as a reference. Cell Ranger also performed sequence identification and correction of individual CDR regions in each BCR sequence. The germline mutations of each cell were calculated using GATK4, and the cells were clustered into five simulated samples via the called mutations using Soupercell.^37^ Comparing the consistency of the mutations in each simulated sample to the mutations in samples of the 3′ RNA and ATAC sequencing data, the cells were ultimately divided into five samples corresponding to the single‐cell 3′ RNA‐seq and ATAC‐seq libraries.

### 4.8 B‐cell clonotype and mutation analysis

The cells were screened and characterised according to the expression profile in the 5′ RNA sequencing data (same as the process for the single‐cell 3′ RNA‐seq described below). The sequences of the CDR3 region were utilised as the BCR signatures and the cell number of each BCR signature type was counted in different B‐cell subtypes. The BCR types with a cell number greater than two were defined as clonal. The mutations in the *IGHV* genes of each cell were calculated by GATK4. The mutation rate of *IGHV* genes was calculated as the proportion of mutated sites in the *IGHV* genes to the length of the gene body in each cell.

### 4.9 Derivation of gene and peak matrices from sequencing data

The raw FASTQ files from scRNA‐seq and scATAC‐seq were processed with ‘cellranger‐arc count’ to align them with the GRCh38 reference genome. The process produced barcoded count matrices of both gene expression and chromatin accessibility data.

### 4.10 Preprocessing and quality control of gene and peak matrices

The raw count matrices for RNA and ATAC for each library were imported into Seurat (version 4.3.0.1).^72^ To detect and eliminate doublets, scDblFinder (version 1.12.0) was utilised, analysing both RNA and ATAC count matrices. Following 10X Genomics quality control guidelines (https://www.10xgenomics.com/cn/analysis‐guides), cells were filtered based on the following criteria: 500–5000 RNA transcript counts, 200–3000 gene counts, 300–50 000 ATAC fragments, a Fragments in Peaks (FRiP) score above 20% (using FRiP function), less than 10% mitochondrial reads (using PercentageFeatureSet function with the ‘MT‐‘ pattern), nucleosome signals below 2 (using NucleosomeSignal function) and a transcription start site (TSS) enrichment above 3 (using TSSEnrichment function) in the Seurat package. Peak calling was performed using macs2 (version 2.2.5). Using mitochondrial sequences and our developed method, MitoSort, we deconvoluted pooled samples and removed doublets resulting from multiple samples.^20^ The parameter used was a p1_cutoff of .8. Based on the aforementioned quality control measures, a total of 54 365 immune cells meeting the selection criteria were obtained. Furthermore, the sex of each sample was annotated using the expression weight of genes in chromosome Y, facilitating the differentiation between patients and healthy controls.

### 4.11 Integrative analysis of single‐cell multi‐omics by weighted nearest neighbour

To begin, rPCA was employed to establish integration anchors based on the RNA modality. The anchors were subsequently applied to align cell embeddings across both RNA and ATAC data. For RNA, this involved running IntegrateData, followed by ScaleData and RunPCA on the integrated assay. For ATAC, IntegrateEmbeddings was executed using ‘lsi’ reductions. Following this, FindMultiModalNeighbors was performed, utilising the integrated RNA data along with the adjusted LSI through the weighted nearest neighbour (WNN) procedure. The integrated dimension reduction approach enabled multimodal clustering (RNA: 1–30, ATAC: 2–50) at a resolution of .5, with uniform manifold approximation and projection (UMAP) used for downscaling and cluster identification. This yielded 21 clusters and identified 16 cell types based on chromatin accessibility in the promoter regions and expression levels of established immune cell marker genes.^73^, ^74^, ^75^

For cell type annotation, we used the following marker genes: naive B cells (CD79A, TCL1A), memory B cells (CD79A and MS4A1), plasma B cells (MZB1, CD27, CD38 and SDC1), naive CD4^+^ T cells (CD4 and CCR7), memory CD4^+^ T cells (CD4 and IL7R), regulatory CD4^+^ T cells (CD4, FOXP3 and PTKN2), naive CD8^+^ T cells (CD8A and CCR7), central memory CD8^+^ T cells (CD8A and GZMK), effector memory CD8^+^ T cells (CD8A and GZMH), mucosal‐associated invariant CD8^+^ T cells (CD8A, GZMK, KLRB1 and NCR3), regulatory CD8^+^ T cells (CD8A, FOXP3 and RTKN2), nature killer (NK) cells (GNLY, NKG7), classical monocytes (VCAN, LYZ and CD14), non‐classical monocytes (MS4A7 and FCGR3A), cDCs (FLT3, FCER1A and CLEC10A) and pDCs (GAS6, IRF8, IRF7 and LILRA4). The transcriptional levels and promoter region accessibility of these genes were visualised using the DotPlot and CoveragePlot functions, respectively, to annotate the identified cell types.

### 4.12 Measurement of correlation coefficients among different samples and cells

To measure transcriptome similarity between cells, we first identified the 2000 genes with the greatest differences in these cells, and then used the expression matrix of these 2000 genes to calculate the Pearson correlation coefficient between cells. The contents of the 16 cell types in each sample were utilised to calculate the similarity between 10 samples by 1 minus the Bray–Curtis distance.

### 4.13 Identifying key genes in a patient's immune system changes

Cell‐type‐specific gene markers were identified using the Logistic Regression (LR) test implemented in the FindAllMarkers function. The significant difference in gene sets between anti‐NMDARE patients and healthy controls in each cell type was identified using the Likelihood Ratio test (nbinomLRT) in Deseq2 package (*Padj *< .01, *|*log2FC*| *> .5).^76^ Then, we employed the correlation‐based package FigR to identify gene‐peak links within a 200 kb range upstream and downstream of the differential gene TSS, exhibiting significant concordance with alterations in the said differential gene (*Padj* < .01). We used the Benjamini–Hochberg method to correct multiple tests. Metascape (https://metascape.org/) was used for enrichment analyses, which encompassed Gene Ontology (GO), Kyoto Encyclopedia of Genes and Genomes (KEGG), Reactome and Wikipathway database.

### 4.14 Processing of transcription factor motifs

TF motifs were obtained from the JASPAR2020 package and yielded 633 motif Pfam matrix. We utilised the FindMotif function for peak‐set analysis to find enriched motifs and ranked them by enrichment score and *p*‐value. Further screening was conducted based on Motif's corresponding gene expression levels and chromVAR activity. To delineate the precise TF binding sites essential for elucidating the gene regulatory network, we utilised Bedtools for extracting peak‐set sequences, then employed the MOODs package for assessing the alignment accuracy between peak sequences and the respective Motif's Pfam matrix. Subsequently, binding peaks of the primary TFs and their associated downstream regulatory genes were ascertained based on the match scores obtained.

### 4.15 Cell–cell communication analysis

To assess intercellular communication, we utilised the CellChat package (release 1.1.3) to explore potential cell interactions, focusing exclusively on secreted signalling ligand–receptor pairs. Briefly, CellChat objects were generated for both patients and healthy controls using the createCellChat function. The probability of communication was then estimated through the computeCommunProb and computeCommunProbPathway functions by identifying genes with over‐expression. Additionally, centrality scores within the network and the impact of each ligand–receptor pair on the signalling pathways were computed using netAnalysis_computeCentrality. The subsetCommunication function was ultimately utilised to obtain the interaction score and *p*‐value for each ligand–receptor pair within each signalling pathway. This process involves validating the expression of each interacting gene to screen for the most credible interactions.

### 4.16 Statistical analysis

The Logistic Regression (LR) test, Likelihood Ratio test and Wilcoxon rank sum test were employed to assess the significance of the gene expression differences between pairs of groups. The one‐way ANOVA test was conducted to compare the cytokine levels among the four groups: acute phase anti‐NMDARE, convalescence anti‐NMDARE, recurrent stage anti‐NMDARE patients and healthy controls. Spearman's correlation coefficient was used to examine the correlation between different cell types, and the Bray–Curtis distance was used to assess the similarity between different individuals. The significance level was established at *p* < .05. For some post hoc analyses, Benjamini–Hochberg correction was applied to adjust for multiple comparisons, maintaining the level of significance at *p* < .05.

## AUTHOR CONTRIBUTIONS

**Yaqing Shu; Jin Xu; Xinhui Li** and **Weixing Zhang** designed and led the project. **Yaqing Shu** provided all the clinical samples and clinical information. **Weixing Zhang** performed tissue collection with help from **Dongjie Peng**. **Weixing Zhang** performed all the single‐cell multi‐omics sequencing libraries and single‐cell immune sequencing libraries. **Dongjie Peng** performed the cytokine release assay. **Huilu Li** performed the Western blot. **Xinhui Li; Yicong Xu; Zihao Chen** and **Zhongjie Tang** analysed and interpreted the data. **Xinhui Li; Yicong Xu; Weixing Zhang; Wenxu Ren** and **Jin Xu** drafted and edited the manuscript; and all the remaining authors commented on the manuscript.

## CONFLICT OF INTEREST STATEMENT

The authors declare they have no conflicts of interest.

## ETHICS STATEMENT

The study adhered to the ethical principles outlined in the Declaration of Helsinki of 1975 and received approval from the local ethics committee of the Third Affiliated Hospital of Sun Yat‐sen University (RG2023‐243‐01). Informed written consent was obtained from all patients or their representatives as well as the healthy control subjects. All experiments and protocols for human studies involving human samples were performed in accordance with the relevant guidelines and regulations.

## Supporting information



Supporting Information

Supporting Information

Supporting Information

Supporting Information

Supporting Information

## Data Availability

The raw sequence data reported in this paper have been deposited in the Genome Sequence Archive^77^ in the National Genomics Data Center,^78^ China National Center for Bioinformation/Beijing Institute of Genomics, Chinese Academy of Sciences (GSA‐Human: HRA004605) and are publicly accessible at https://ngdc.cncb.ac.cn/gsa‐human. The Code used for data analysis and visualisation is available at https://github.com/lixh257/anti‐NMDARE.
