# Supplementary material for: Immunoregulatory programs in anti‐N‐methyl‐D‐aspartate receptor encephalitis identified by single‐cell multi‐omics analysis
Source: Clin Transl Med. 2025 Jan 8;15(1):e70173. doi: 10.1002/ctm2.70173 (PMC11710936; doi:10.1002/ctm2.70173)
Supplement: Supplementary file 1 — Supporting Information [file CTM2-15-e70173-s003.docx]

**Supplemental figures and legends**


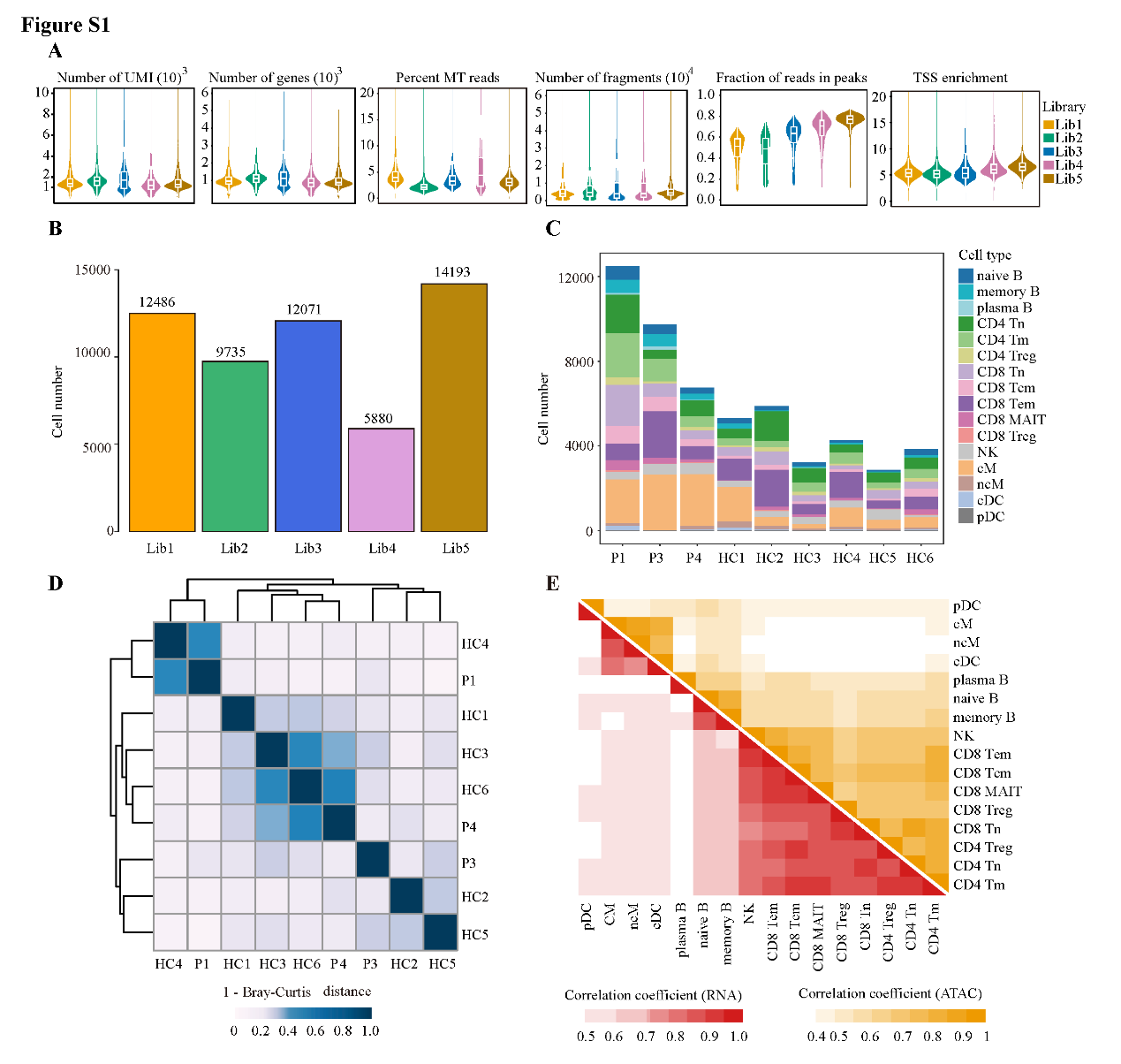


**Figure S1**. **Quality control of single-cell multi-omics datasets**.

(A) Violin plot illustrating the distribution of RNA/ATAC quality metrics across samples, encompassing the count of unique molecular identifiers (UMIs), gene count, and the proportion of mitochondrial (MT) reads for each library in single-cell RNA sequencing (scRNA-seq). For single-cell ATAC sequencing (scATAC-seq), the plots depict the number of unique fragments, fraction of reads in peaks, and TSS enrichment score.

(B) Bar plot displaying the number of cells post-quality control in the five libraries.

(C) Stacked bar graph illustrating the distribution of 16 cell types in the nine samples.

(D) Heatmap representing the inter-sample distances. Color intensity corresponds to 1 minus the Bray-Curtis dissimilarity index. Darker shades indicate closer relationships.

(E) Heatmap showing the Pearson correlation coefficients between cell types, calculated using matrices derived from transcriptome and chromatin accessibility data.


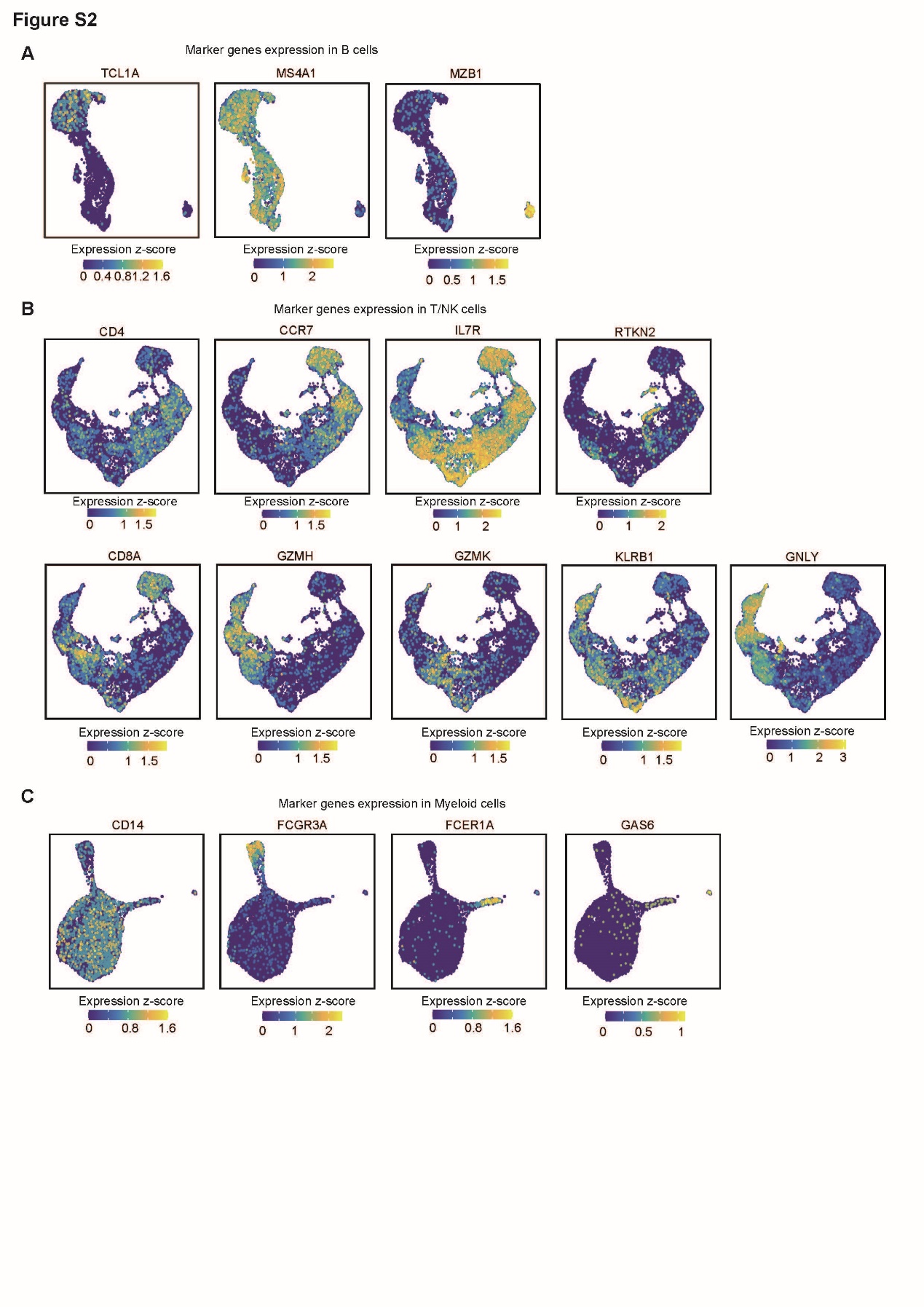


**Figure S2. Marker gene expression in UMAPs.**

(A−C) UMAP plots were utilized to delineate the expression distribution of marker genes indicative of cell types, with color intensity reflecting the normalized expression levels. Panels A, B, and C correspond to B cells, T cells, and myeloid cells, respectively.


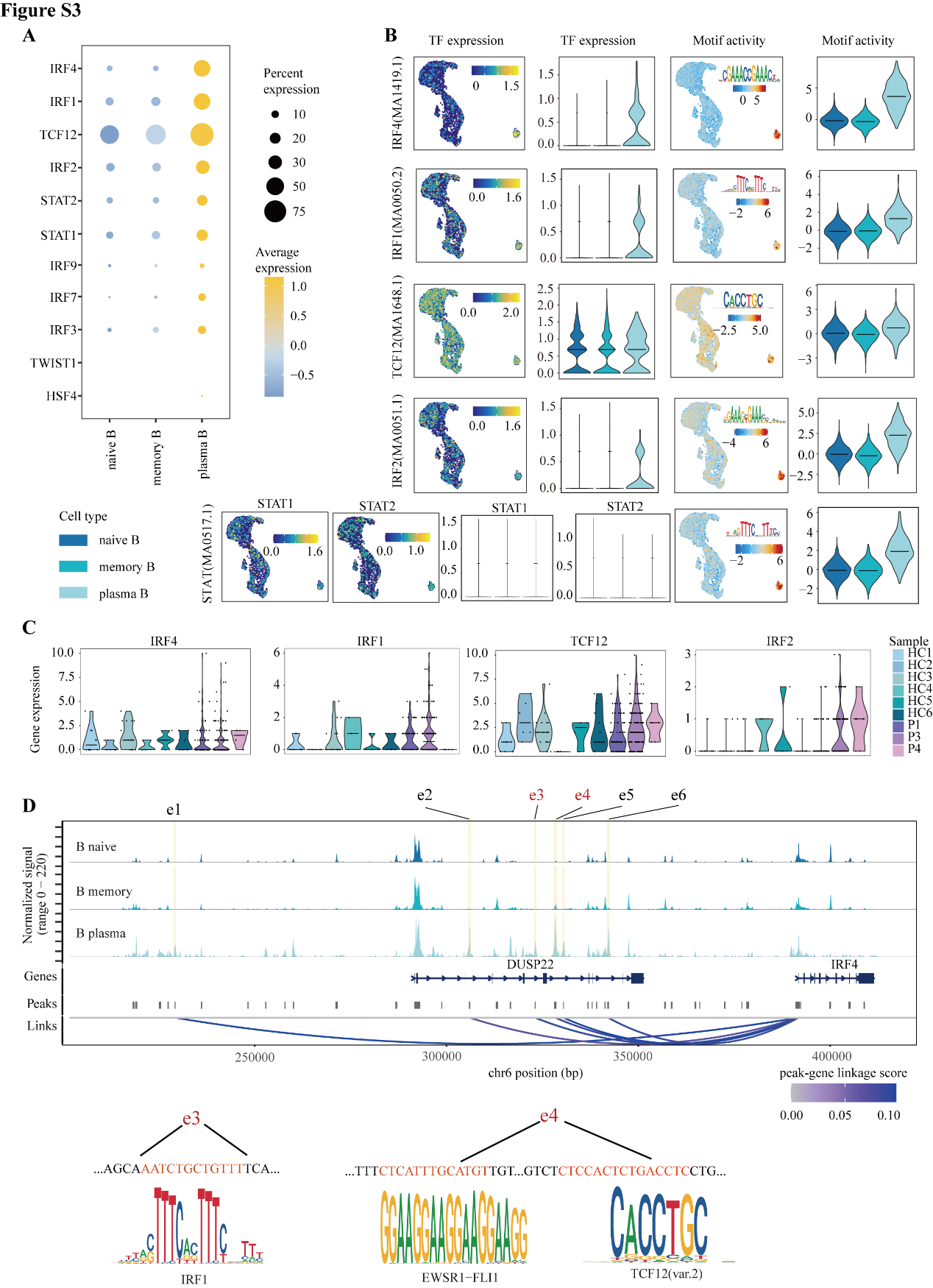


**Figure S3. Gene expression level in plasma B cells.**

(A, B) Expression levels of TFs enriched in B cells were depicted using a bubble plot (A) and violin plots, along with a UMAP visualization (B), to showcase the chromVAR activity across the naive B, memory B, and plasma B cells.

(C) Violin plot displaying the expression levels of four key TFs in plasma B cells across the nine samples, providing a detailed view of the distribution and density of the expression data.

(D) Genome track showing peaks with motifs that are positively linked to IRF4 expression. Tracks from top to bottom are normalized chromatin accessibility around the IRF4 gene in naive B, memory B, plasma B cells, gene annotation, peaks, and peak-to-gene links. The enhancer regions of IRF4 with high chromatin accessibility in plasma B cells, which are linked to IRF4 expression levels, are highlighted in light orange.


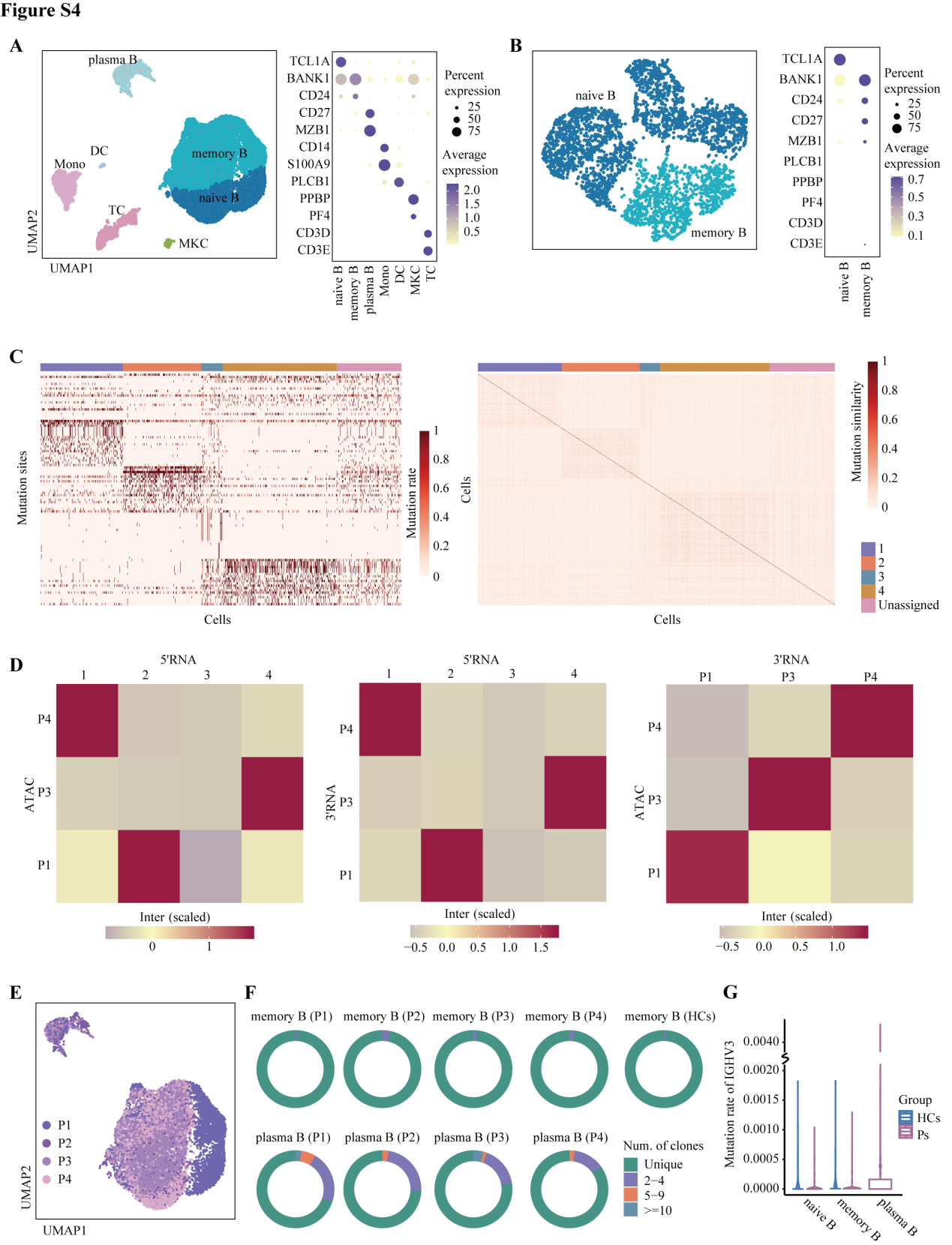


**Figure S4. Cellular heterogeneity and mutational profiles in disease- and normal-state of B cells.**

(A) UMAP representation of all scRNA-seq cells passing quality control, colored by annotated clusters. Broad cell types are labeled on UMAP (left). scRNA gene expression for selected marker genes for each cluster in patients. Color indicates relative expression across all clusters and dot size indicates the percentage of cells in that cluster expressing the gene (right).

(B) UMAP representation of memory and plasma B cells passing quality control, colored by annotated clusters (left). scRNA gene expression for selected marker genes for each cluster in HCs. Color indicates relative expression across all clusters and dot size indicates the percentage of cells in that cluster expressing the gene (right).

(C) Analysis of characteristic mutational hotspots within each cell from diseased samples. The left heatmap presents the frequency of detected germline mutation sites from 5' RNA-seq across cells, with the horizontal axis denoting individual cells and the vertical axis indicating mutation sites. The right heatmap depicts the frequency similarity of these mutations between cells within each separated sample.

(D) Comparative analysis of germline mutation sites across different sequencing modalities derived from the same data sets of samples. The color gradient represents the degree of consistency (ratio of consistent mutations, scaled). The left panel compares ATAC-seq with 5' RNA-seq, the middle panel compares 3' RNA-seq with 5' RNA-seq, and the right panel compares ATAC-seq with 3' RNA-seq.

(E) A UMAP showing the cell distribution of each patient in the 5' RNA sequencing data.

(F) A ring graph depicting the number of clonotypes per sample and cell type. These numbers were divided into four levels, indicated by different colors.

(G) Mutation rates of the IGHV3 gene in naive B cells, memory B cells, and plasma B cells, with colors indicating whether the cells originate from anti-NMDARE patients or healthy controls sourced from public resources.


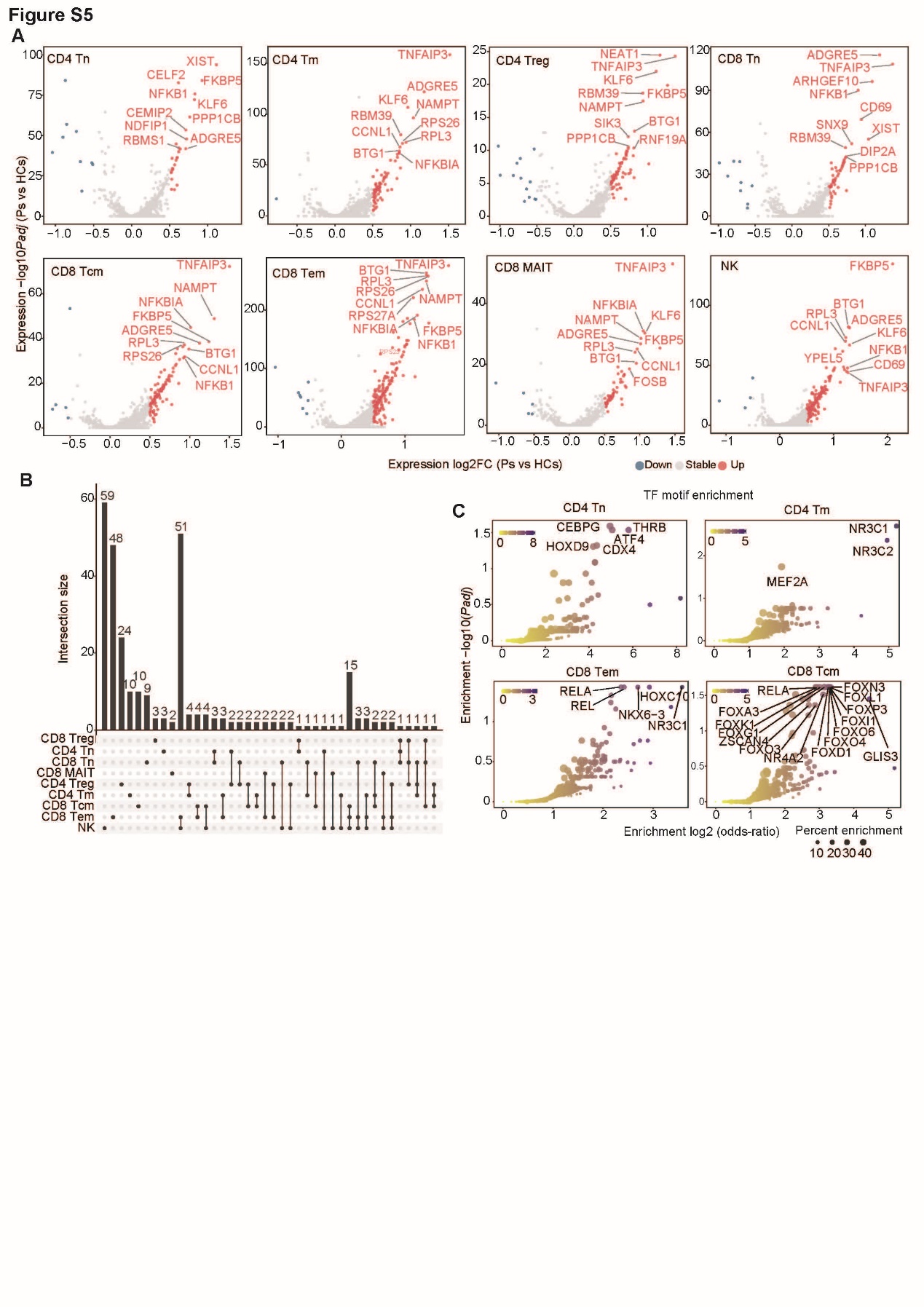


**Figure S5. Differentially expressed genes in T cells.**

(A) A Volcano plot delineating the DEGs within differential cell types of T cells comparing Ps with HCs. Upregulated genes in Ps are highlighted in red and the top 10 gene symbols are labeled.

(B) An Up-Set plot visualizing the intersecting distribution of DEGs across various cell types.

(C) A dot plot displaying the enrichment results of several significantly enriched motifs and the significantly enriched motifs are marked.


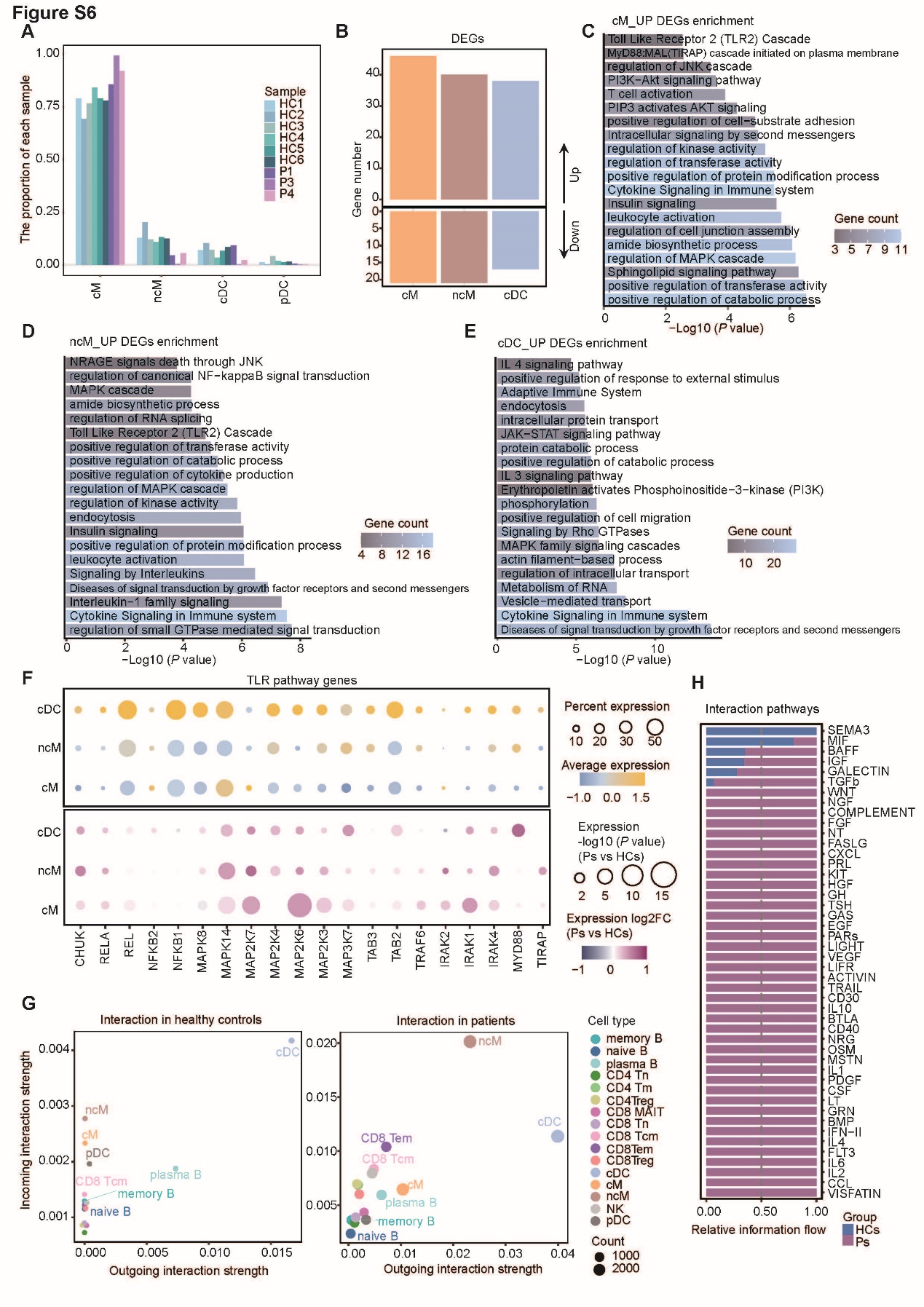


**Figure S6. Functional characteristics of myeloid cells.**

(A) A bar plot displaying the myeloid cellular proportion across the nine samples.

(B) A bar plot displaying the number of DEGs between Ps and HCs in three myeloid cell types.

(C–E) Bar plots illustrating the enriched functional terms and pathways of upregulated genes specific to cM (C), ncM (D), and cDC (E) cell types within the patient cohort.

(F) Expression levels and differential expression of downstream genes of the TLR2 pathway across cM, ncM, and cDC cell types.

(G) A two-dimensional bubble plot depicting the intensity and quantity of interactions involving different immune cells between patient and healthy control groups.

(H) A bar plot representing the normalized interaction profiles between patients and healthy controls across distinct pathways.


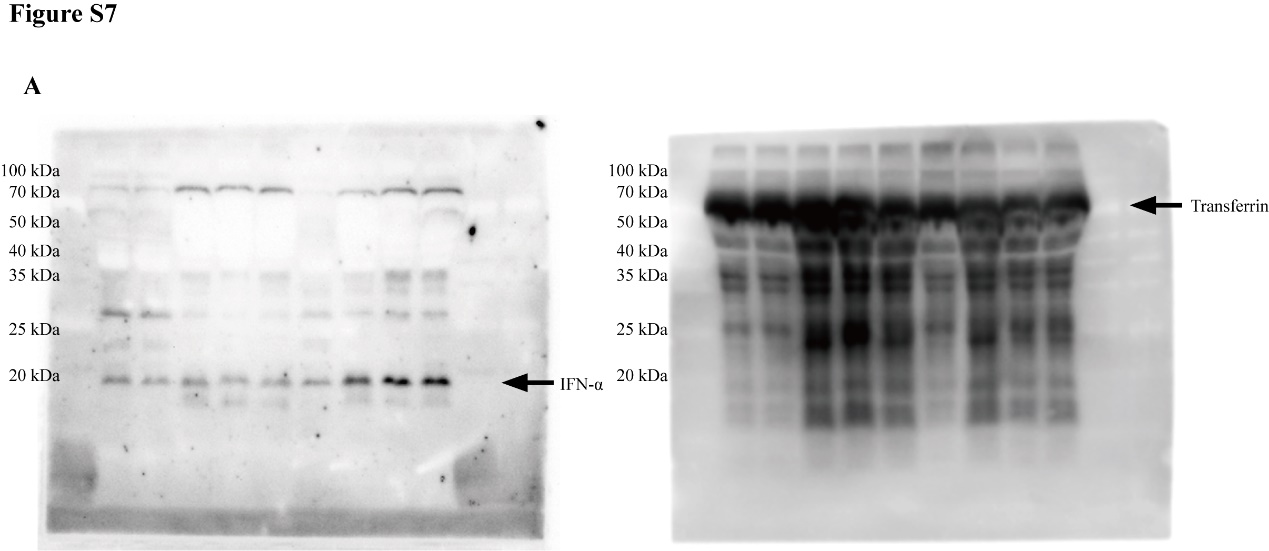


**Figure S7. The IFN-α levels in humanized mice model.**

1. The levels of IFN-α in the serum of humanized mice engrafted with PBMCs from anti-NMDARE patients (Patient), healthy donors (HC), and medium-only controls (NC) were assessed using Western blot analysis. Each group comprised three samples.

**
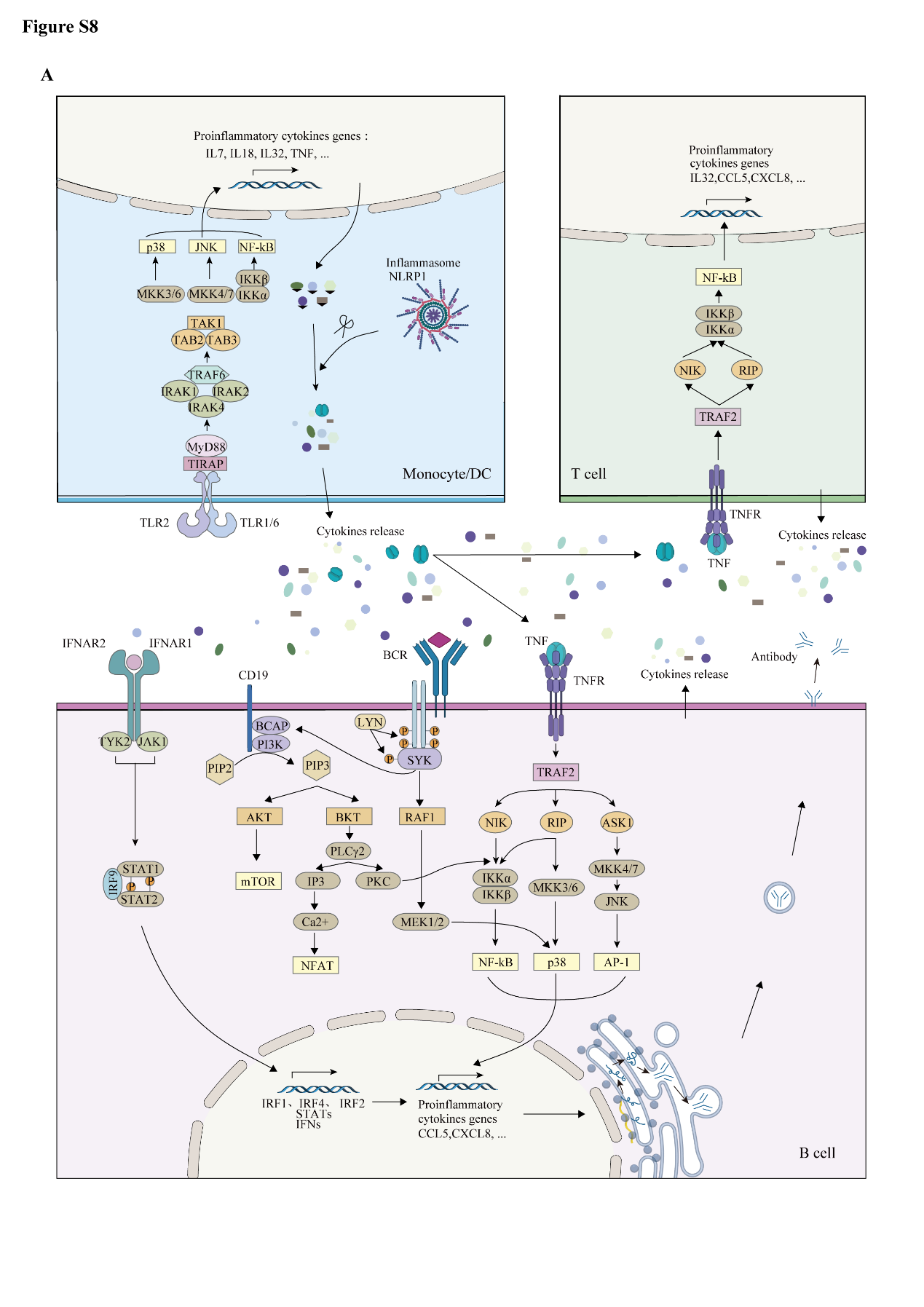
**

**Figure S8. Immune cell regulatory patterns in PBMCs of patients with anti-NMDARE.**

1. A regulatory model diagram of different immune cells in PBMCs from anti-NMDARE patients and the cell-cell communications.


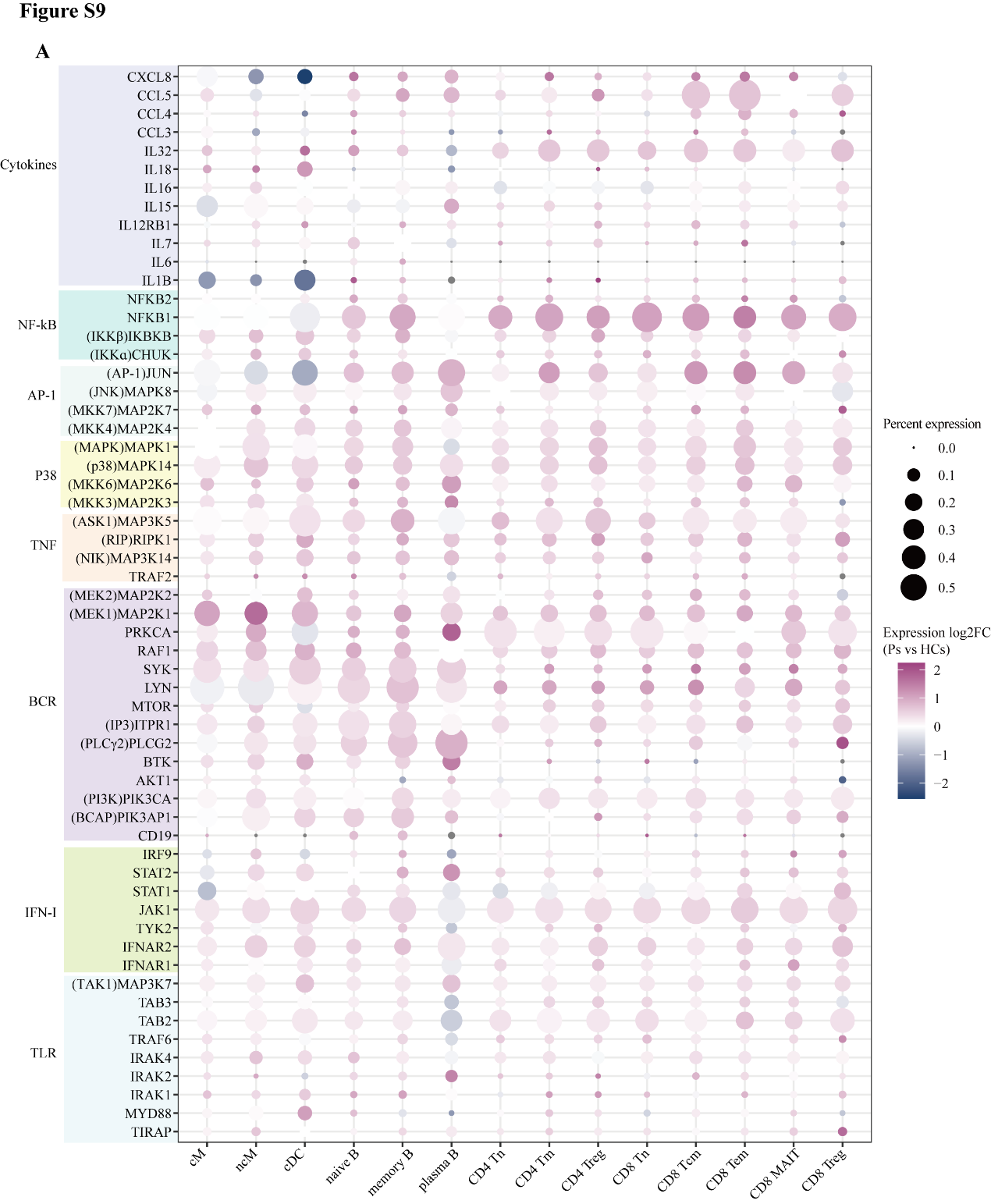


**Figure S9. Differences in expression levels of immunoregulatory pathway genes in anti-NMDARE patients**

(A) A dot plot displaying gene expression in **immunoregulatory** pathways of different cell types.

**Supplemental tables and legends**

**Table S1. Sample information (related to table 1).**

| Sample | | Library | Gender | Age |
| --- | --- | --- | --- | --- |
| P1 | Lib1 | | Female | 24 |
| P2 | Lib1 | | Male | 23 |
| P3 | Lib2 | | Male | 18 |
| P4 | Lib3 | | Female | 18 |
| HC1 | Lib3 | | Male | 24 |
| HC2 | Lib4 | | Male | 24 |
| HC3 | Lib5 | | Male | 24 |
| HC4 | Lib5 | | Female | 22 |
| HC5 | Lib5 | | Female | 22 |
| HC6 | Lib5 | | Female | 22 |

**Table S2.** Excel file containing additional data too large to fit in a PDF, related to the function enrichment results in figure 2F.

**Table S3.** Excel file containing additional data too large to fit in a PDF, related to the linkage information in the TF-gene regulation network in figure 2I.

**Table S4.** Excel file containing additional data too large to fit in a PDF, related to the linkage information in the TF-gene regulation network in figure 4E.

**Table S5.** Excel file containing additional data too large to fit in a PDF, related to the function enrichment results in figure 4F.
